# Characterization and evaluation of the therapeutic benefits of pure and lanthanides mono- and co-doped zinc oxide nanoparticles

**DOI:** 10.1016/j.sjbs.2023.103608

**Published:** 2023-02-23

**Authors:** Maryam Al Bitar, Bahaa Hassanieh, R. Awad, Mahmoud Khalil

**Affiliations:** a Department of Physics, Faculty of Science, Beirut Arab University, Beirut, Lebanon; b Department of Biological Sciences, Faculty of Science, Beirut Arab University, Beirut, Lebanon; c Molecular Biology Unit, Department of Zoology, Faculty of Science, Alexandria University, Alexandria, Egypt

**Keywords:** Lanthanides-doping, Co-precipitation, Antibacterial activity, Cytotoxic effect, Cancer

## Abstract

The effect of Lanthanides-doping on the structural, optical, morphological, antibacterial and anticancer properties of zinc oxide (ZnO) nanoparticles was investigated. Pure ZnO, Zn_0.9_La_0.1_O, Zn_0.9_Ce_0.1_O, and Zn_0.9_La_0.05_Ce_0.05_O were fabricated through the chemical co-precipitation route. The structural and morphological properties were studied using the X-ray diffraction (XRD) and transmission electron microscopy (TEM), respectively. The optical properties were analyzed by photoluminescence spectroscopy (PL). The inhibitory effect of the synthesized nanoparticles (NPs) was assessed against six bacterial strains using the agar well diffusion and broth micro-dilution methods. The anticancer potential of the synthesized NPs was assessed against two human colon cancer cell lines Caco-2 and HCT-116. The appearance of the La_2_O_3_ and CeO_2_ secondary phases upon doping La^3+^ and Ce^3+^ ions induced structural and morphological changes. The large distorted hexagonal morphology of pure ZnO is transformed into small sized distorted hexagonal form. The photoluminescence spectra revealed the point defects resulting from Lanthanum (La) and cerium (Ce) doping. The prepared NPs significantly inhibited the growth of the six investigated bacteria and induced cytotoxic effects and morphological changes against Caco-2 and HCT-116 cell lines. This study showed that doping ZnO with lanthanide ions such as La^3+^ and Ce^3+^ provide promising biological applications. These NPs showed a potent antibacterial and anticancer effect towards the investigated bacterial strains and colon cancer cell lines. These findings point to the importance of the biological applications of NPs, and the possibility of investigating other biomedical applications for NPs.

## 1 Introduction

Nanotechnology is the study of manipulating and controlling matter on the nanoscale level utilizing logical insight into different biotechnological applications (Jeevanandam et al., 2018). A wide range of nanoscale materials with unique physical, chemical, optical and magnetic properties have recently been developed in this field (Khan et al., 2019). Considering these unique properties, NPs can be used in several biomedical, industrial, healthcare, and environmental applications (Khan et al., 2019). In particular, metal and metal oxide NPs have garnered considerable attention due to their highly potent antibacterial and anticancer effects (Dizaj et al., 2014, Hamida et al., 2020a, Vinardell and Mitjans, 2015). ZnO NPs exhibited the best bactericidal effect towards different bacterial strains and induced higher toxicity against various cancer cell lines than other metal and metal oxide NPs (Azam et al., 2012, Abbott Chalew and Schwab, 2013). In addition, ZnO NPs are of special relevance for their therapeutic benefits as antibacterial and anticancer agents (Jiang et al., 2018). Although ZnO NPs possess remarkable properties which make them suitable for various technological applications, these NPs showed limited stability in biological applications due to their instability in water (Lallo da Silva et al., 2019). Thus, doping can significantly enhance the physical, chemical, photocatalytic, antibacterial, and anticancer properties of ZnO Nps (Carofiglio et al., 2020, Lallo da Silva et al., 2019). In particular, doping with lanthanides is vital due to their distinctive features (Lee et al., 2017, Wen and Wang, 2014, Nabeel, 2020). In this context, La-doped ZnO nano-collides have exhibited substantial inhibiting activity against a set of bacteria and brine shrimps due to morphology changes, including shape modification and a decrease in the NPs’ size (Shahzad et al., 2019). On the other hand, the high concentration of Ce present in Ce-doped ZnO samples possessed more inhibitory activity toward bacteria than the lower dopant concentration (Bomila et al., 2017). In addition, the simultaneous doping of La^3+^ and Ce^3+^ ions into the ZnO matrix induced a better inhibitory effect on a set of bacteria when compared with the simultaneous doping of either La^3+^ or Ce^3+^ ions with gadolinium ion (Gd^3+^) (Bomila et al., 2019). Furthermore, lanthanide ions (La^3+^, Ce^3+^, and Neodymium (Nd^3+^))-doped ZnO NPs exhibited various degrees of toxicity against A498 cells with minimal toxicity towards normal Vero cells and showed a potent inhibitory effect (Karthikeyan et al., 2019). Until now, the mechanism by which NPs inhibited bacteria and induced cytotoxic effects is still poorly understood. Different studies have proposed that the cellular interaction of NPs enhances the reactive oxygen species (ROS) production, which affects the structure of DNA, enzymes, and lipids and may result in cell damage (Hamida et al., 2020c, Hamida et al., 2020b, Ullah et al., 2022). Many techniques have been used for the synthesis of lanthanides-doped ZnO NPs, such as co-precipitation (Theivarasu and Indumathi, 2017), sonochemical (Phuruangrat et al., 2016), gel combustion (Nguyen et al., 2019), and biological synthesis (Karthikeyan et al., 2018a). The co-precipitation method is used in the present work since it is a cost-effective method, in addition to its simplicity, better control of particle morphology and composition, and easy control of the pH and sintering temperature (Almoussawi et al., 2020). In this context, it was reported that doping ZnO with lanthanide ions, synthesized via the precipitation method, enhanced the antibacterial and anticancer potential compared with pure ZnO NPs (Manikandan et al., 2017, Nabeel, 2020, Theivarasu and Indumathi, 2017). Based on the literature, few studies have reported the biological activity of lanthanides mono- and co-doped ZnO NPs. On the other hand, capping agents can significantly influence the morphology and stability of the NPs throughout the synthesis process (Chandrasekaran et al., 2012, Javed et al., 2016). Ethylenediamine tetra acetic acid (EDTA) is particularly relevant since it has high capping suitability on the surface of NPs, allowing the NPs to be highly water-dispersible, and can be used as a stabilizer (Jang et al., 2018, Nithiananth and Velraj, 2016, Yi et al., 2014). Knowing that EDTA comprises six reaction sites, four of which are hydroxyl groups that can form coordination bonds with Zn^2+^ ions and so efficiently affect the morphology of the NPs during crystal formation (Chandrasekaran et al., 2012).

The purpose of this work is to evaluate the biological activity of pure ZnO, Zn_0.9_La_0.1_O, Zn_0.9_Ce_0.1_O, and Zn_0.9_La_0.05_Ce_0.05_O synthesized using the chemical co-precipitation technique and capped with EDTA. The characterization of the synthesized NPs was studied using the XRD, TEM, and PL analysis. The antibacterial potential of the synthesized NPs was investigated towards six bacterial strains including *Escherichia coli*, *Citrobacter braakii*, *Klebsiella pneumonia*, *Staphylococcus aureus*, *Streptococcus intermedius*, and *Staphylococcus haemolyticus*. In addition, the antitumor effect of these NPs was assessed towards two human colon cancer cell lines (Caco-2 and HCT-116).

## 2 Materials and methods

### 2.1 Chemicals and reagents

Zinc chloride (ZnCl_2_, ≥98.0%) (HiMedia, India), lanthanum (iii) chloride (LaCl_3_, 99.9 %) (Alfa Aesar, ThermoFisher, Germany). Cerium (iii) chloride heptahydrate (CeCl_3_·7H_2_O, ≥98.0%), Sodium hydroxide (NaOH, ≥98.0%), EDTA (C_10_H_14_N_2_O_8_Na_2_·2H_2_O), Dulbecco’s Modified Eagle’s Medium–high glucose (DMEM), Fetal bovine serum (FBS), Dulbecco’s Phosphate Buffered Saline (PBS), Thiazolyl Blue Tetrazolium Bromide powder (MTT), and crystal violet (Sigma-Aldrich, Germany). Mueller-Hinton agar (MHA), Nutrient Broth (NB), and Nutrient Agar (NA) (Scientific Technical Supplies).

### 2.2 Fabrication process

The chemical co-precipitation process was used for the fabrication of pure ZnO (Al Bitar et al., 2022), Zn_0.9_La_0.1_O, Zn_0.9_Ce_0.1_O, and Zn_0.9_La_0.05_Ce_0.05_O. An adequate amount of ZnCl_2_, LaCl_3_, CeCl_3_·7H_2_O, and 0.1 M EDTA were weighed and then dissolved in distilled water as a dispersing solvent. The solutions were then titrated by 4 M NaOH, added in drop-wise with constant stirring, until pH reached 12. The solutions were then heated for 2 h at 60 ℃ with constant stirring, and washed several times with distilled water until the pH reduced to 7. The obtained white precipitates were then dried at 100 ℃ for 18 h, ground and calcinated at 550 ℃ for 4 h. From now on, pure ZnO, Zn_0.9_La_0.1_O, Zn_0.9_Ce_0.1_O, and Zn_0.9_La_0.05_Ce_0.05_O will be referred to as ZnO-Pure, ZnO-La, ZnO-Ce, and ZnO-LaCe, respectively.

The prepared samples were characterized by X-ray Diffraction (XRD) using Bruker D8 Focus powder diffractometer, with *Cu*$K_{\alpha }$ radiation (*λ* = 1.54056 Å) in the range of 25° ≤ 2*θ* ≤ 80°. The surface morphology of the prepared NPs was measured using Transmission electron microscope (TEM) JEM-1400 Plus. Using the photoluminescence spectrophotometer FP-8600 with an excitation wavelength of 325 nm and a wavelength ranges from 350 to 700 nm, the emission spectra of the synthesized NPs were examined.

### 2.3 Cell culture

Two human colon cancer cell lines Caco-2 and HCT-116 were purchased from the American Type Culture Collection (ATCC) (Manassas, VA, USA). Cells at the 8–12 passages were used in the experiments. The cell lines were cultured in DMEM culture medium supplemented with 10 % FBS, 1 % Penicillin-Streptomycin antibiotic (biowest, France), and 1 % l-Glutamine (Sigma-Aldrich, Germany). The cells were incubated in a humidified atmosphere (5 % CO_2_ and 37 ℃).

### 2.4 MTT assay

100 μl of culture medium containing 5 × 10^5^ /ml of HCT-116 or Caco-2 cells were seeded to each well of 96-well plate. DMEM culture medium was used as the vehicle for the prepared NPs, and six replicates were considered for each concentration (0.1–3.2 mM). Wells having culture medium only were considered as a negative control. Cells were incubated for 24 h in order for the cells to adhere to the plate bottom (5 % CO_2_ and 37 ℃). Cells were incubated with increasing concentrations of the NPs for 24–48 h. Following the incubation period, 10 μl of 5 mg/ml MTT solution was added to the cells, and the cells were incubated for 3–4 h. After labeling the cells with MTT, as described above, the solution was removed and 50 µl of isopropyl alcohol solution was added to each well and mixed thoroughly, then incubated for additional 10 min at 37 °C. Subsequently, the plates’ light absorption was measured at 540 nm in a microplate (ELISA) reader.

The cell viability (%) was calculated by the following equation:

Cell viability (%) = ((mean treated cells OD-blank OD)/ (control OD-blank OD)) × 100.

### 2.5 Cell morphological analysis and crystal violet staining

5 × 10^5^/ml HCT-116 or Caco-2 cells were seeded in 12-well plates, cultured in DMEM complete media, and then incubated for 24 h (5 % CO_2_ and 37 ℃). Cells were incubated with increasing concentrations of the NPs (0.1–1.6 mM) for 24–48 h. The morphological changes were visualized, examined, and images were captured using Leica DM500 microscope and compared with control untreated cells.

### 2.6 Antibacterial activity of the NPs

#### 2.6.1 Microbial strains and antibacterial activity of the synthesized NPs using the agar well diffusion method

Six bacteria were used for the assessment of the antibacterial activity of the prepared NPs. These bacteria included gram-negative (*Escherichia coli*, *Citrobacter braakii*, and *Klebsiella pneumonia*) and gram-positive (*Staphylococcus aureus*, *Streptococcus intermedius*, and *Staphylococcus haemolyticus*) bacteria. *Escherichia coli*, *Citrobacter braakii*, *Staphylococcus aureus*, *Staphylococcus haemolyticus*, and *Streptococcus intermedius* were isolated from the waste water collected from a station related to South Lebanon Water Establishment (SLWE) in Sidon city and *Klebsiella pneumonia* obtained from Mount Lebanon Hospital (UMC). The identification of the isolated bacteria was made using the VITEK analysis. The same experimental procedure was done as discussed and illustrated by Al Bitar et al. (2022) to assess the inhibitory effect of the synthesized NPs (200, 100, 50, and 25 mg/ml) against the six investigated bacteria. Serial half-fold dilutions of the three antibiotics (AB; Ciprofloxacin, Amoxicillin, and Doxycycline) (12.5 mg/ml, 6.25, 3.125, and 1.5625 mg/ml) and sterile distilled water were used. The zone of inhibition (ZOI) was measured and presented as the mean ± the standard error of the mean (SEM) after incubating the plates for 24 h at 37 ℃. Methicillin Resistance test was done on the two *staphylococcus* specie*s* (*Staphylococcus haemolyticus* and *Staphylococcus aureus*). Resistance to Cefoxitin (30 µg) indicated that the tested *Staphylococcus* species are Methicillin Resistance (Figure S1).

#### 2.6.2 Minimum inhibitory concentration (MIC)

In order to quantify the antibacterial activity, the MIC values of the prepared NPs were estimated using the broth micro-dilution method using 96-well U-bottom microtiter plates. Bacterial suspension of the six bacteria was done by transferring the fresh colonies grown overnight on NA plates to sterile test tubes containing nutrient broth. Serial half-fold dilution of the NPs was prepared by dispersing the NPs in nutrient broth. The test was carried out by distributing 100 μl per well bacterial suspension with final inoculum concentrations of 10^6^ CFU/ml (Iseppi et al., 2020). Then, this 100 μl per well bacterial suspension was treated with 100 μl of the prepared NPs dilutions (triplicate) to achieve the desired concentrations ranging between 200 and 0.01221 mg/ml. After 24 h, the MIC was determined by measuring the optical density (OD) at 590 nm. The OD values for the wells with concentrations ranging between 12.5 and 0.01221 mg/ml were registered and plotted.

#### 2.6.3 Minimum bactericidal concentration (MBC)

The MBC was determined after broth micro-dilution by sub-culturing the different concentrations ranging between 200 mg/ml − 0.01221 mg/ml from wells on MHA plates. The plates were then incubated for 24 h at 37 °C and observed for bacterial growth (Jam et al., 2022).

### 2.7 Statistical analysis

The obtained data were represented as the mean ± SEM of at least three independent replicates, and two-way ANOVA was used for the statistical analysis using the GraphPad Prism 6 program (Ver. 6.01). *p* values were calculated such that *p* > 0.05 was not significant (ns), *p < 0.05, ^**^p < 0.01, and ^***^p < 0.001, where *, **, and *** denote levels of significance.

## 3 Results

### 3.1 Structural and optical analysis

**Fig. 1:**
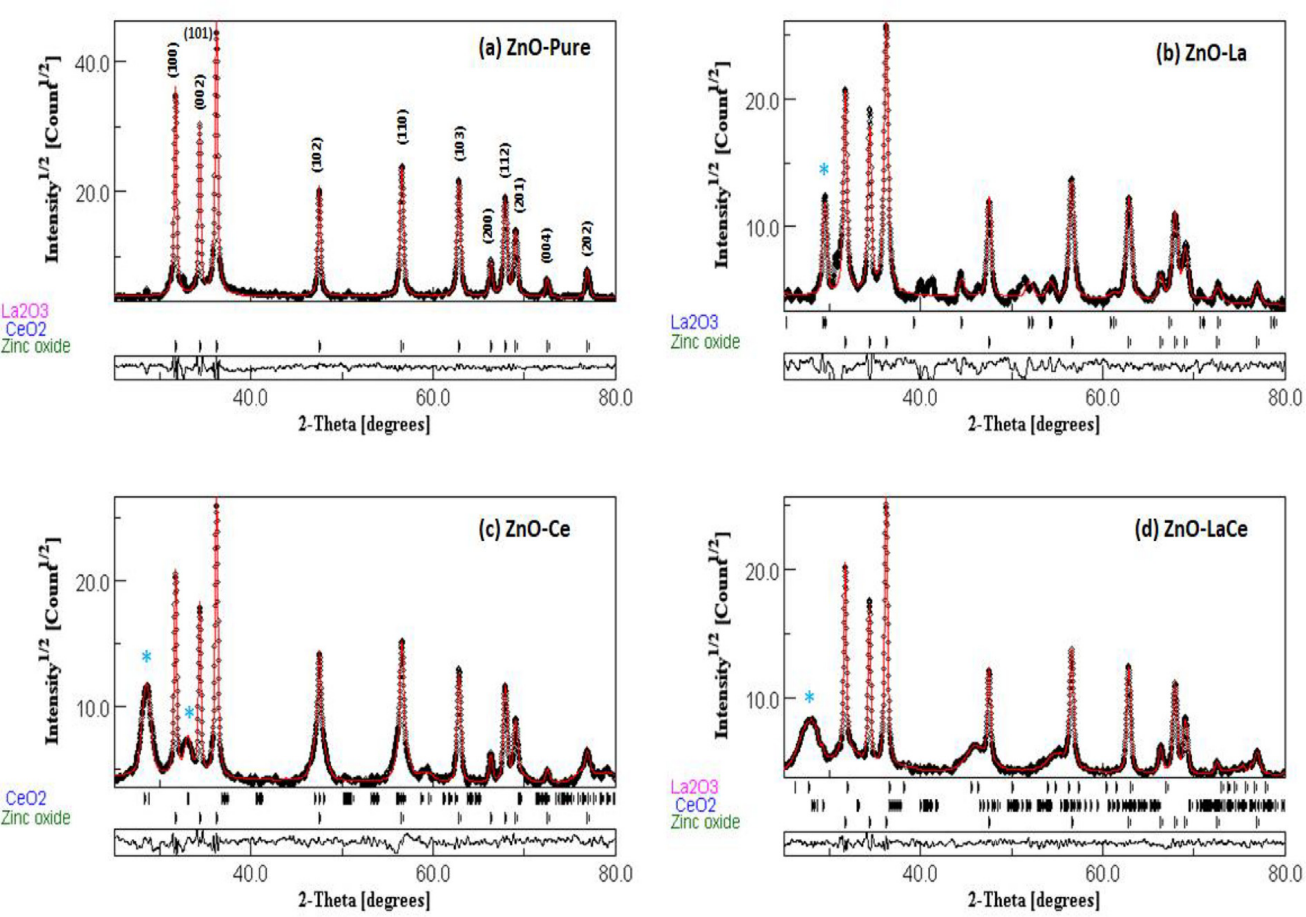
X-ray diffractogram patterns with their corresponding Rietveld refinements of ZnO-Pure, ZnO-La, ZnO-Ce and ZnO-LaCe NPs performed on the MAUD software. Note: *, Secondary peaks.

To examine the purity of the ZnO phase, the Maud program was used for the structural Rietveld refinements of the X-ray diffractogram patterns of ZnO-Pure (Al Bitar et al., 2022), ZnO-La, ZnO-Ce, and ZnO-LaCe (Fig. 1). These patterns are indexed to the ZnO wurtzite structure with space group *P*6_3_*mc* and elven main diffraction peaks, corresponding to the crystalline planes (1 0 0), (0 0 2), (1 0 1), (1 0 2), (1 1 0), (1 0 3), (2 0 0), (1 1 2), (2 0 1), (0 0 4), and (2 0 2), respectively (AL-Asady et al.., 2020, Almoussawi et al., 2020, Kumar and Sahare, 2014, Morkoç and Özgür, 2009). In addition, the detection of secondary peaks in the obtained patterns is due to the doping with La^3+^ and/or Ce^3+^ ions. These secondary peaks correspond to the appearance of secondary phases as La_2_O_3_ in the ZnO-La sample, CeO_2_ in the ZnO-Ce sample, and both La_2_O_3_ and CeO_2_ phases in the ZnO-LaCe sample (Fig. 1). The percentages of the secondary phases present in the synthesized NPs, and the refined lattice parameters *a* and *c* values with their corresponding ratios $\left(c/a\right)$ obtained from the Maud program are listed in Table 1. The values of the refined lattice parameters show a slight change upon doping. ZnO-La and ZnO-LaCe samples exhibit lower *a* values, whereas the ZnO-Ce sample exhibits higher *a* value compared to the ZnO-pure sample. Furthermore, the three doped samples register lower *c* values compared to the ZnO-pure sample. In order to determine the deviation of the crystal lattice from the perfect arrangement, the degree of distortion (R) is calculated using equation (1) (Yasmeen et al., 2020). The obtained $\left(c/a\right)$ ratios and the degree of distortion (R) stay almost constant around (1.60) and (1.019), respectively.

**Table 1:** Structural parameters of ZnO-Pure (Al Bitar et al., 2022), ZnO-La, ZnO-Ce and ZnO-LaCe NPs obtained from XRD refinement and TEM.

Samples Parameters	ZnO-Pure	ZnO-La	ZnO-Ce	ZnO-LaCe
a (Å)	3.2529	3.2525	3.2530	3.2524
c (Å)	5.2116	5.2075	5.2104	5.2108
c/a	1.6021	1.6011	1.6017	1.6021
R	1.0193	1.0199	1.0195	1.0193
ZnO %	100.0000	97.5730	79.9629	77.5499
CeO_2_ %	0.0000	0.0000	20.0371	2.1787
La_2_O_3_ %	0.0000	2.4262	0.0000	20.2713
D_DSM_ (nm)	33.8439	22.3807	28.9080	28.3184
D_TEM_ (nm)	70.6144	29.4324	7.2347	72.4369/10.5671

On the other hand, the average crystallite size (D_DSM_) values are calculated using the Debye –Scherrer model (DSM), given by equation (2) (Farhat et al., 2018):

(1) $$R=\sqrt{\frac{8}{3}}\times \frac{a}{c}$$

(2) $$D_{\mathrm{DSM}}=\frac{k\lambda }{\beta_{\mathrm{hkl}}\cos\theta_{\mathrm{hkl}}}$$

where k, λ, $\beta_{hkl}$ and θ represent the shape factor constant (0.9), *Cu*$K_{\alpha }$ X-ray wavelength, full width at half maximum and peak position, respectively. $D_{DSM}$ values are computed from the plot’s slope, fitted according to equation (2). The obtained average crystallite size decreases upon doping with La^3+^ and/or Ce^3+^ ions (Table 1).

**Fig. 2:**
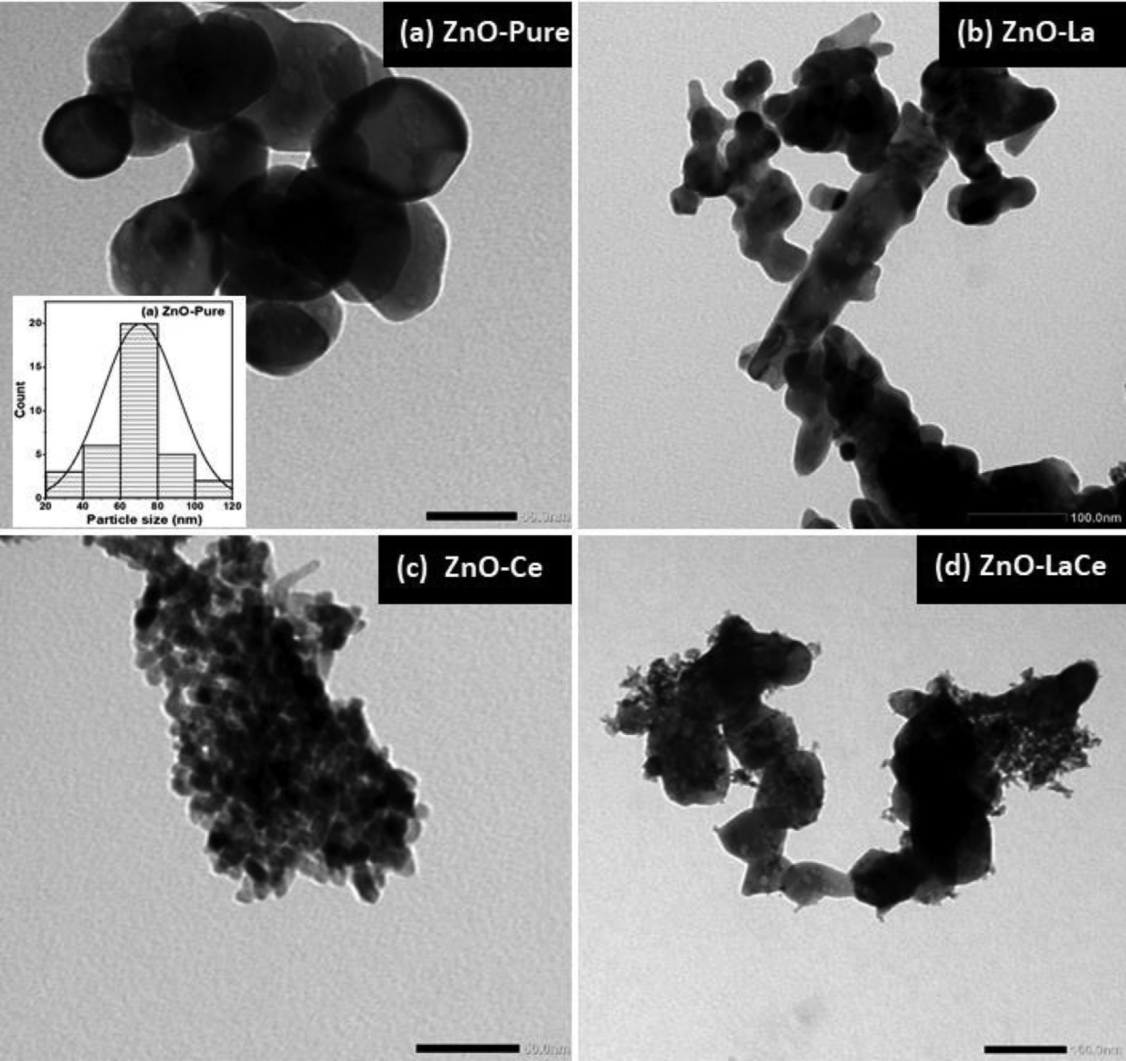
TEM images of (a) ZnO-Pure (Al Bitar et al., 2022), (b) ZnO-La, (c) ZnO-Ce and (d) ZnO-LaCe NPs. The inset of (a) shows the average particle size distribution histogram for ZnO-Pure sample (Al Bitar et al., 2022). The specified scales (a) 50 nm, (b)100 nm, (c) 50 nm, and (d) 100 nm.

Non-agglomerated NPs are seen in the TEM images with different average sizes and distorted hexagonal shapes (Fig. 2). The average particle size (D_TEM_) of the synthesized NPs is obtained from the particle size distribution histogram, as shown in Fig. 2(a). ZnO-Pure NPs demonstrate a distorted hexagonal shape having an average particle size of 70.614 nm (Al Bitar et al., 2022). The influence of doping with La^3+^ and/or Ce^3+^ ions on ZnO NPs evident, as shown in Figures (2b, c, and d). The average particle size decreases to 29.43 nm with La doping, whereas it decreases more to 7.23 nm with Ce doping (Table 1). However, the simultaneous doping of La^3+^ and Ce^3+^ ions in the ZnO-LaCe sample shows two distinguishable distorted hexagonal shapes with two average particle sizes of 72.44 and 10.56 nm. The D_TEM_ values obtained from the particle size distribution histograms showed insignificant variation compared to the D_DSM_ values calculated using the Debye –Scherrer model (DSM), as shown in Table 1.

**Fig. 3:**
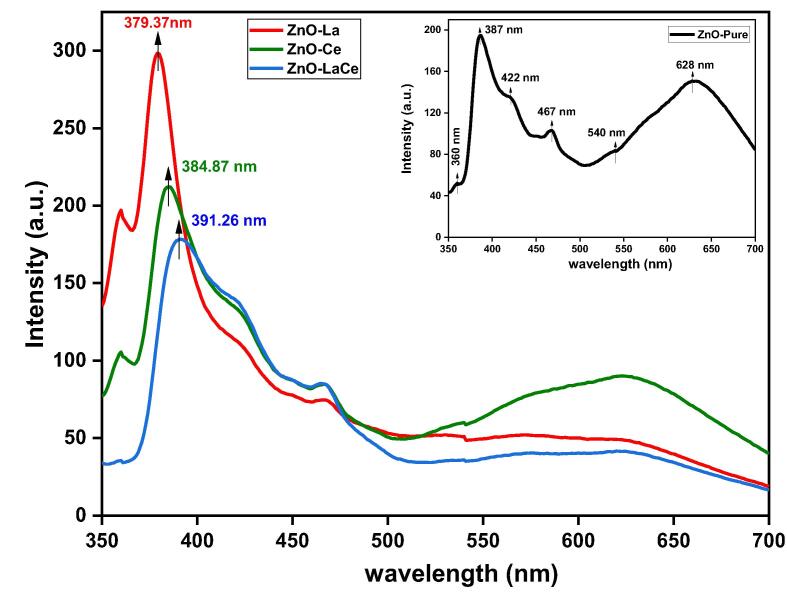
Photoluminescence emission spectra of ZnO-Pure (Al Bitar et al., 2022), ZnO-La, ZnO-Ce and ZnO-LaCe NPs.

The PL spectra of ZnO-Pure (Al Bitar et al., 2022), ZnO-La, ZnO-Ce, and ZnO-LaCe samples were examined in the range of 350–700 nm, as displayed in Fig. 3. All the samples demonstrate two UV emission peaks corresponding to the near band edge emission (NBE). Peak located at 387 nm is shifted to a lower wavelength value with La or Ce doping in ZnO-La and ZnO-Ce samples, whereas it is shifted to a higher wavelength value with the simultaneous doping of La^3+^ and Ce^3+^ ions in the ZnO-LaCe sample. In the UV region, the ZnO-LaCe sample registers the lowest intensity compared to other samples (Fig. 3). However, La doping results in a remarkable increase in the intensity of the NBE peaks, which means La^3+^ ions induce more excitons (Obeid et al., 2019). Furthermore, the emission peaks corresponding to deep-level (DL) emission can be attributed to several structural defects. These include oxygen or zinc vacancies, oxygen or zinc interstitial, or oxygen antisites present in the samples (Mishra et al., 2010). Accordingly, the intensity of the peaks due to DL emission in the visible region decreased upon incorporating La^3+^ and/or Ce^3+^ ions (Fig. 3).

### 3.2 Antibacterial activity

**Fig. 4:**
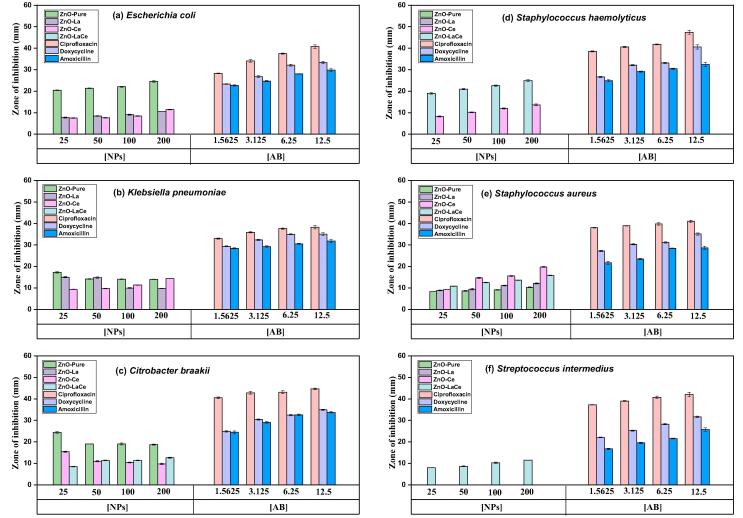
Inhibitory effect of ZnO-Pure (Al Bitar et al., 2022), ZnO-La, ZnO-Ce and ZnO-LaCe, Ciprofloxacin, Doxycycline and Amoxicillin toward six bacteria, (a) *Escherichia coli*, (b) *Klebsiella pneumonia*, (c) *Citrobacter braakii*, (d) *Staphylococcus haemolyticus*, (e) *Staphylococcus aureus*, and (f) *Streptococcus intermedius*. Results are represented as mean ± SEM of at least three experiments. **Abbreviation:** SEM, standard error of the mean.

The inhibitory effect of ZnO-Pure (Al Bitar et al., 2022), ZnO-La, ZnO-Ce, and ZnO-LaCe samples was tested against six bacterial strains (*Escherichia coli*, *Citrobacter braakii, Klebsiella pneumoniae*, *Staphylococcus aureus*, *Streptococcus intermedius*, and *Staphylococcus haemolyticus*) using the agar well diffusion and broth micro-dilution methods. The calculated ZOI results of the synthesized NPs with the standard antibiotics (Ciprofloxacin, Doxycycline, and Amoxicillin) are presented in Fig. 4 and listed in Table S1. The existence of an inhibition zone demonstrates the inhibitory effect of the standard antibiotics and the prepared samples (Figures S2, S3, and S4). The confidence interval, *p* values, and the significance level are listed in Tables S2, S3, and S4. As the concentration of the three used antibiotics increases, the inhibitory effect increases (Fig. 4). Among the used antibiotics, Ciprofloxacin registered the highest antibacterial activity against the investigated bacteria. *Escherichia coli*, *Citrobacter braakii*, and *Klebsiella pneumoniae* were the most bacteria sensitive to ZnO-Pure NPs, as reported previously by Al Bitar et al. (2022). However, both ZnO-La and ZnO-Ce samples inhibited the growth of both *Escherichia coli* and *Klebsiella pneumonia* to a lesser extent, and no inhibition was noticed by the ZnO-LaCe sample against these two bacteria. Furthermore, ZnO-Ce and ZnO-LaCe samples inhibited the growth of *Citrobacter braakii*. In addition, both ZnO-Pure and ZnO-La samples showed inhibition inversely proportional to the increase in NPs’ concentration against *Klebsiella pneumonia*. This behavior was also seen by the ZnO-Pure and ZnO-Ce samples against *Citrobacter braakii*. On the other hand, ZnO-Pure and ZnO-La samples showed no antibacterial effect against the *Staphylococcus haemolyticus*; however, ZnO-Ce and ZnO-LaCe samples inhibited the growth of this bacterium. In contrast, the three doped samples enhanced the antibacterial activity of the ZnO-Pure sample toward *Staphylococcus aureus*. Only the simultaneous doping of La^3+^ and Ce^3+^ ions in the ZnO-LaCe sample showed a minimal inhibitory effect against *Streptococcus intermedius*; the ZOI decreased from 11.47 ± 0.05 to 8.03 ± 0.07 mm with the decrease in the concentration of the NPs from 200 to 25 mg/ml.

The MIC values were determined for the samples which inhibited bacterial growth using the agar well diffusion method. The graphical presentation of the obtained OD values outcome is highlighted in Figures S5 and S6. The minimum concentrations corresponding to the lowest OD values are listed in Table 2. *Escherichia coli* was highly sensitive to ZnO-Pure NPs with an MIC value of 1.563 mg/ml, compared to ZnO-La and ZnO-Ce, with an MIC of 3.125 mg/ml (Table 2). On the other hand, *Citrobacter braakii* was the most bacterium responsive to ZnO-Pure NPs with an MIC value of 1.563 mg/ml, whereas it showed a higher MIC value of 6.250 mg/ml for ZnO-Ce and ZnO-LaCe samples (Table 2). However, *Klebsiella pneumonia* was found to be most susceptible to the ZnO-La sample and registered the lowest MIC value of 0.391 mg/ml compared to ZnO-Pure and ZnO-Ce samples with 3.125 and 6.250 mg/ml, respectively (Table 2). In contrast, *Staphylococcus aureus* and *Staphylococcus haemolyticus* were highly responsive to the simultaneous doping of La^3+^ and Ce^3+^ ions with low MIC values of 0.391 and 0.781 mg/ml, respectively (Table 2). In addition, in the present study, all the samples showed no minimum bactericidal concentration (MBC).

**Table 2:** The minimum inhibitory concentration (MIC) of ZnO-Pure, ZnO-La, ZnO-Ce and ZnO-LaCe NPs.

Sample	Bacteria	MIC (mg/ml)
ZnO-Pure	*Escherichia coli*	1.563
	*Klebsiella pneumoniae*	3.125
	*Citrobacter braakii*	1.563
	*Staphylococcus aureus*	1.563
ZnO-La	*Escherichia coli*	3.125
	*Klebsiella pneumoniae*	0.391
	*Staphylococcus aureus*	3.125
ZnO-Ce	*Escherichia coli*	3.125
	*Klebsiella pneumoniae*	6.250
	*Citrobacter braakii*	6.250
	*Staphylococcus aureus*	1.563
	*Staphylococcus haemolyticus*	3.125
ZnO-LaCe	*Citrobacter braakii*	6.250
	*Staphylococcus aureus*	0.781
	*Staphylococcus haemolyticus*	0.391
	*Streptococcus intermedius*	1.563

### 3.3 Anticancer activity

**Fig. 5:**
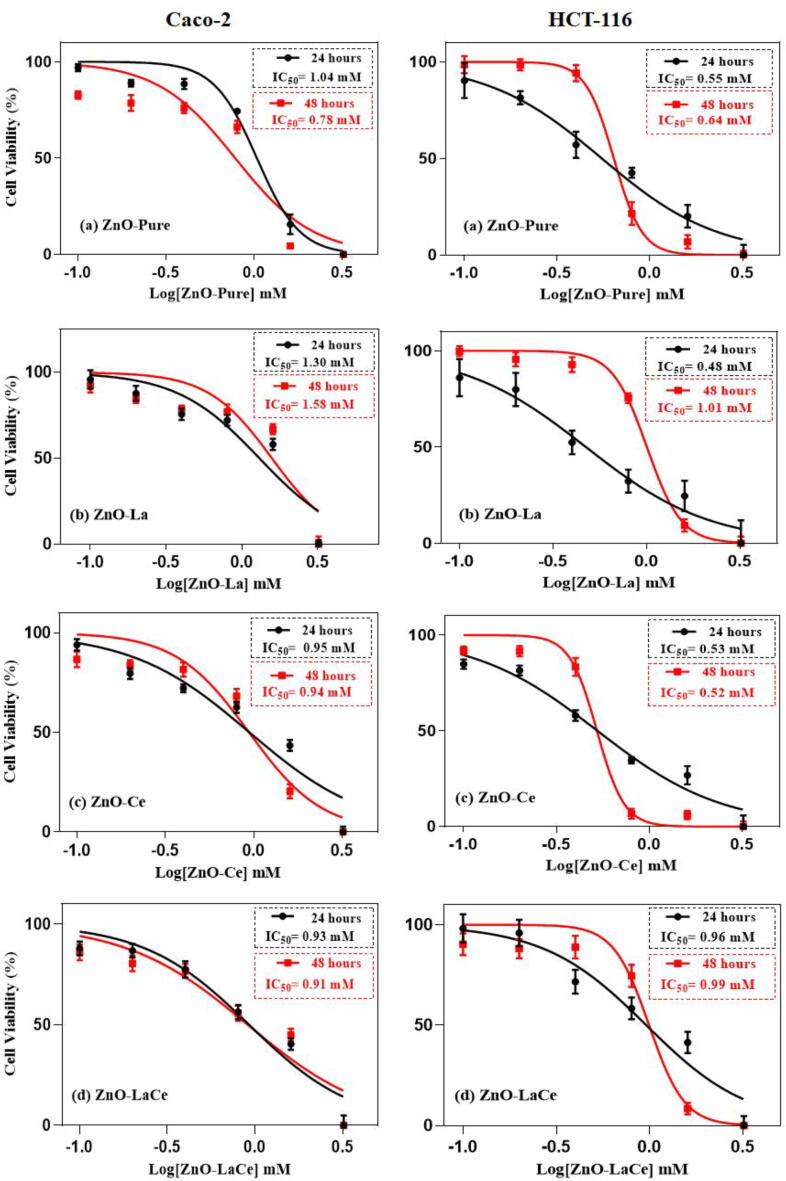
Effect of ZnO-Pure, ZnO-La, ZnO-Ce and ZnO-LaCe NPs on the cell viability of Caco-2 and HCT-116 cells. MTT assay was performed to detect the living cells after 24 or 48 h, respectively. The IC_50_ values were calculated after treating the cells with varying concentrations of the prepared NPs. The obtained data are represented as mean ± SEM of six independent replicates. **Abbreviation:** SEM, standard error of the mean.

The anticancer potential of the synthesized samples was evaluated against Caco-2 and HCT-116 cell lines using the MTT assay. The IC_50_ values were calculated after treating the cells with increasing concentrations of the NPs (0.1–3.2 mM) for 24–48 h. At 24 h, ZnO-LaCe and ZnO-La samples showed the highest toxicity against Caco-2 and HCT-116 cells compared to the other prepared samples with IC_50_ values 0.93 and 0.48 mM, respectively. At 48 h, the calculated IC_50_ value against Caco-2 cell line after exposing the cells to ZnO-Pure treatment was 0.78 mM; however, doping ZnO with La^3+^ and/or Ce^3+^ ions induced less toxicity against this investigated cell line and showed higher IC_50_ values, such that the calculated IC_50_ values for ZnO-La, ZnO-Ce, and ZnO-LaCe samples were 1.58, 0.94, and 0.91 mM, respectively (Fig. 5). Furthermore, doping with Ce enhanced the cytotoxic effect of ZnO-Pure NPs against HCT-116 cell line (IC_50_ = 0.52 mM) for 48 h. The obtained data showed that the synthesized NPs induced a significant decrease in the proliferation of the cells in a dose- and time-dependent manner compared to the untreated control cells at concentrations above 0.4 mM (Figure S7).

**Fig. 6:**
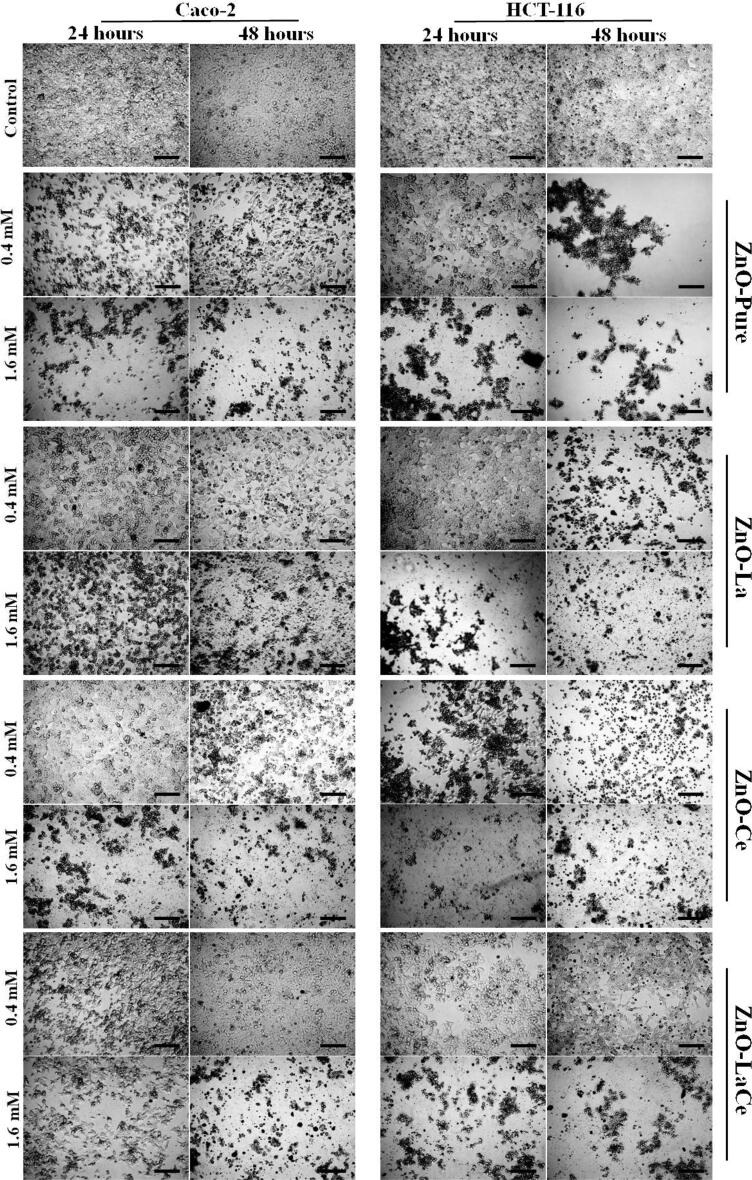
Morphological changes induced by ZnO-Pure, ZnO-La, ZnO-Ce and ZnO-LaCe NPs with varying concentrations on Caco-2 and HCT-116 cells after 24–48 h using the Inverted Microscope with × 200 magnification with scale bar value 100 µm.

The cell morphological changes were examined for both treated cell lines with the synthesized NPs concentrations (0.1–1.6 mM) after 24 and 48 h (Fig. 6, Figures S8, S9, S10, and S11). No significant difference in morphology was seen between the control and the treated cells at low concentrations (0.1 and 0.2 mM). However, at higher concentrations (0.4, 0.8, and 1.6 mM), the treated cells showed remarkable alteration in normal morphology, cell shrinkage, and size reduction. The reduction of the viable cells was observed in the treated cells by staining the cells with crystal violet solution (Figure S12). This verified the MTT assay results of cell proliferation and cytotoxicity induced by the synthesized NPs.

## 4 Discussion

The obtained X-ray diffractogram patterns with their corresponding Rietveld refinements reveal the fingerprint of the hexagonal wurtzite structure (Fig. 1). The formation of the secondary phases in the three doped samples can be linked to the larger ionic radii of La^3+^ and Ce^3+^ ions compared to Zn^2+^ ions, preventing the substitutional incorporation of La and/or Ce in Zn-sites and causing deposition on the ZnO surface (Ahmad et al., 2015, Bomila et al., 2019; Suwanboon et al., 2013). The change in the *a* and *c* values may be due to the formation of the secondary phases or the mismatch of the ionic radii of the dopants La^3+^ and Ce^3+^ ions and host Zn^2+^ ions (Iqbal et al., 2009; Suwanboon et al., 2013). The calculated R values are greater than unity (R > 1), indicating crystal lattice distortion (Yasmeen et al., 2020). The constant values of c/a ratios and R indicate that doping with La^3+^ and/or Ce^3+^ ions does not cause a considerable impact on the wurtzite hexagonal structure of the ZnO host lattice (Bomila et al., 2017, Labhane et al., 2018). The particle size and degree of crystallinity of the three doped samples decreased, as indicated by the XRD peaks broadening with the decrease in their intensities (Pal et al., 2012, Poornaprakash et al., 2016). A Similar phenomenon was observed by Ahmad et al. (2015) in the case of doping ZnO NPs with Ce ions. Ahmad et al. (2015) suggested that the localization of the dopant ions in or near the boundary of ZnO NPs may reduce the diffusion rate and hinder the NP growth, resulting in a decrease in their crystallite size. The morphology “size and shape” of ZnO-Pure NPs was modified via doping with La^3+^ and/or Ce^3+^ ions and using EDTA as a capping agent (Fig. 2). As reported in the literature, the NP morphology of doped ZnO with La^3+^ and/or Ce^3+^ ions showed either a spherical or rod shape (Bomila et al., 2019, Goel et al., 2017, Jayachandraiah et al., 2014). Similarly, it was reported that other lanthanides (Ln^3+^ = Eu^3+^, Gd^3+^, Sm^3+^, Pr^3+^, and Er^3+^)-doped ZnO-NPs, fabricated without using a capping agent, exhibited nanorod, irregular, and non-uniform morphologies (Chen et al., 2018, Farhat et al., 2018, Nabeel, 2020). However, in the present study, the NP shape prepared via the co-precipitation method was controlled using EDTA as a capping agent, and a distorted hexagonal form was obtained. The estimated values of D_TEM_ are different from the D_DSM_ values obtained using the XRD studies, as displayed in Table 1. ZnO-Pure and ZnO-La NPs registered higher D_TEM_ values compared to the estimated D_DSM_ values. For D_TEM_ greater than D_DSM_, this difference can be attributed to the accumulation of crystals in the TEM technique due to grain growth, as reported by Kamareddine et al. (2020). As a result, particles composed of several crystallites are formed, each corresponding to a coherent diffraction domain. However, it can be assigned to the reaction solution aging in the case of D_TEM_ smaller than D_DSM_, as reported by Bitar et al. (2017). In the present case, the particle size is highly affected by the aging in the reaction solution, particularly in ZnO-Ce and ZnO-LaCe samples, due to the doping with Ce^3+^ ions.

The PL spectra of all the synthesized samples exhibited both UV and DL emission peaks (Fig. 3). The UV emission peaks are due to the recombination of electron-hole pairs corresponding to the NBE transition of ZnO NPs (Lang et al., 2010, Sharma and Jha, 2017). It was reported that the strain induced in the crystal lattice via doping might be responsible for the shift in the UV emission peak (Pandey et al., 2015). The decrease in the PL intensity in the UV region could imply a lower electron-hole recombination rate, leading to higher photocatalytic activity (Flores-Carrasco et al., 2021, Lang et al., 2016). In addition, the quenching of the peaks due to DL emission in the visible region may be caused by photo-generated electrons being trapped in the trap centers (Limaye et al., 2011, Patel et al., 2017). It was reported that the visible color emissions could be useful for cell labeling applications (Tang et al., 2010). Moreover, the presence of defects and oxygen vacancies play a crucial role in enhancing the antibacterial effects (Bomila et al., 2017).

The synthesized samples demonstrated significant antibacterial activity on the investigated bacteria (Fig. 4). ZnO-Pure NPs inhibited the growth of the three tested gram-negative bacteria and staphylococcus *aureus*. In comparison with the three doped samples, ZnO-Pure NPs have shown the highest antibacterial activity towards *Escherichia coli*, *Citrobacter braakii*, and *Klebsiella pneumonia* and showed a small inhibitory effect against Staphylococcus *aureus.* Doping ZnO with either La^3+^ or Ce^3+^ ions caused a small suppression of *Escherichia coli* and *Klebsiella pneumonia* growth compared to ZnO-Pure NPs. However, doping with both dopant ions did not exert antibacterial activity toward these bacteria. Even though ZnO-La and ZnO-Ce inhibited the growth of *Escherichia coli* and *Klebsiella pneumonia* to a lesser extent than ZnO-Pure NPs, these NPs showed better inhibitory effect towards these bacteria compared with other metal oxides NPs and La or Ce mono-doped ZnO NPs (Gnanam et al., 2019, Karthikeyan et al., 2018b, 2019). The growth of *Citrobacter braakii* was suppressed slightly following treatment with ZnO-Ce and ZnO-LaCe NPs due to the presence of Ce^3+^ ions. Only doping with La^3+^ ions prevented the inhibition of this bacterium. Even though the tested *Staphylococcus aureus* was methicillin-resistant, all the prepared NPs fabricated using the co-precipitation method inhibited its growth. In this context, two studies were reported by Bomila et al. (2017, 2019) investigating the antibacterial effect of mono- and co-doped ZnO NPs with rare-earth ions fabricated using the wet chemical method. In the first study, ZnO-Pure and ZnO-Ce samples showed no inhibitory effect towards *Staphylococcus aureus* at low concentrations and small inhibition against this bacterium at high concentrations (Bomila et al., 2017). In the second study, ZnO-Pure and the rare earth-doped ZnO NPs, including ZnO-LaCe samples, did not register an antibacterial effect against the tested *Staphylococcus aureus* (Bomila et al., 2019). Similarly, in the three studies conducted by Karthikeyan et al. (2018a, 2018b, 2019) the tested rare earth mono-doped ZnO NPs, fabricated using the green method, registered a minimal inhibitory effect against *Staphylococcus aureus.* Thus, the co-precipitation method used in this study is more beneficial than the fabrication methods mentioned in the literature. On the other hand, the growth of *Staphylococcus haemolyticus* was significantly inhibited only after treatment with ZnO-Ce and ZnO-LaCe NPs. In this study, among the three doped samples, ZnO-Ce and ZnO-LaCe NPs registered the highest antibacterial activity against *Staphylococcus aureus* and *Staphylococcus haemolyticus*, respectively*.* On the other hand, *Streptococcus intermedius* was the most bacterium that resisted the inhibitory action of ZnO-Pure, ZnO-La, and ZnO-Ce samples. Our results revealed that the simultaneous doping of La^3+^ and Ce^3+^ ions improved the antibacterial activity against this bacterium. Similarly, it was reported by Karthikeyan et al. (2018a) that the inhibition of bacterial growth was achieved by the ZnO-LaCe sample against the investigated bacteria; however, gram-positive bacteria were more responsive to the effect of doping.

So far, the precise inhibitory action of NPs toward bacterial cells remains unclear. Some studies have shown that doping with La^3+^ and/or Ce^3+^ ions enhanced the inhibitory effect of ZnO NPs towards a set of bacterial strains (Karthikeyan et al., 2018a, Karthikeyan et al., 2018b, Manikandan et al., 2017, Theivarasu and Indumathi, 2017). The authors attributed this enhancement activity to different factors, including the increase in dopants concentration, decrease in the particle size, and the release of La^3+^, Ce^3+^, and Zn^2+^ ions. In our study, the variation in the antibacterial activity could be attributed to the difference in the morphology of the synthesized NPs, as shown in the TEM images (Fig. 2). This behavior could also be attributed to the appearance of the secondary phases obtained in the X-ray diffractogram patterns and verified by the Maud program (Fig. 1) In addition, the interaction of the synthesized NPs with the bacterial cell wall may be impacted by the presence of defects and oxygen vacancies (Fig. 3). Based on the broth micro-dilution and agar well diffusion methods, the ZnO-Pure sample was highly responsive to *Escherichia coli* and *Citrobacter braakii*. However, the MIC value of the ZnO-La sample against *Klebsiella pneumonia* was found to be lower than those of the ZnO-Pure and ZnO-Ce samples. Furthermore, the MIC values registered by the ZnO-LaCe sample toward the two *Staphylococcus* species were lower than those of the rest investigated samples. Even though ZnO-Ce NPs registered higher MIC values when compared to ZnO-Pure, ZnO-La, and ZnO-LaCe NPs against the investigated bacteria, these MIC values did not exceed 6.250 mg/ml. In contrast, the MIC of Ce-doped ZnO NPs observed by Fifere et al. (2021) against both *Staphylococcus aureus* and *Escherichia coli* was 20 mg/ml, which was higher than that obtained by the present study. Accordingly, the synthesized samples inhibited the bacterial growth without inducing bacterial cell death. Thus, in the present study, the samples had a bacteriostatic effect only.

In the current study, Caco-2 and HCT-116 cell lines were chosen to evaluate the cytotoxicity effect of the synthesized NPs. Herein, the cytotoxic effect was examined by measuring the cell viability (%) of the treated cells with increasing concentrations of the NPs (0.1–3.2 mM) using the MTT assay for 24–48 h (Fig. 5). Various degrees of toxicity were induced by the prepared samples against the two human colon cell lines. The estimated IC_50_ values were obtained in the range of 0.48–1.58 mM. Based on the obtained IC_50_ values, ZnO-Pure and mono-doped ZnO NPs with either La^3+^ or Ce^3+^ ions exhibited more cytotoxicity towards HCT-116 cells than Caco-2 cells for both tested periods. In contrast, the obtained data revealed that substituting Zn^2+^ ions with La^3+^ and Ce^3+^ ions exerted nearly the same cytotoxic effect toward both examined cells. The sensitivity of Caco-2 cells towards ZnO-LaCe and ZnO-Pure treatments for 24 and 48 h, respectively, was higher than that of HCT-116 cells. However, HCT-116 cells were more responsive to ZnO-La and ZnO-Ce treatments for 24 and 48 h, respectively. In addition, the treated cells with concentrations (≥ 0.4 mM) showed alterations in normal morphology and cell adhesion capacity compared to control cells (Fig. 6). For instance, Jasim and Saleh (2019) reported that ZnO NPs induced cytotoxicity and morphological changes in HCT-116 cells in a dose-dependent manner. Majeed et al. (2019) showed that bio-synthesized ZnO NPs induced significant cytotoxicity against HCT-116 cells with relatively less toxicity toward Vero cells. Similarly, El-Belely et al. (2021) verified that ZnO NPs reduced the proliferation of Caco-2 cells, whereas less toxicity was induced against normal lung cells. Furthermore, a recent study conducted by Aljohar et al. (2022) showed that chemically synthesized ZnO NPs exerted significant cytotoxic effect on human A431 skin carcinoma cells and displayed much lower toxicity to normal kidney Vero cells. This could be explained by the higher metabolic rates of cancer cells and thus their increased demand for nutrient acquisition, rendering them more sensitive than normal cells when exposed to the same treatments (Hammoudi et al., 2011, Ren et al., 2022). Sanaeimehr et al. (2018) showed that ZnO-pure NPs, synthesized using the green method, caused in killing 50 % of human liver cancer cells (HepG2) at concentrations greater than 87 μg/ml at 150 μg/ml after treating the cells for 24, 48, and 72 h. Similarly, Boroumand Moghaddam et al. (2017) reported that the green-synthesized ZnO-NPs induced an anticancer activity against breast cancer cells (MCF-7) with IC_50_ equal to 121 μg/ml after exposing the cells for 24 h. In addition, Dulta et al. (2021) reported that green-synthesized ZnO-Pure NPs induced cytotoxicity in human cervical cancer cells (HeLa) and human colon cancer cells (HT-29) after 24 h with IC_50_ values of 101.7 μg/mL and 124.3 μg/ml, respectively. On the other hand, Theivarasu and Indumathi. (2017) found that at 280 ± 0.05 μg/ml, chemically synthesized ZnO-Pure NPs using the co-precipitation method killed 50 % of (A549) human lung cancer cells. Compared with this mentioned literature, ZnO-Pure NPs fabricated in the present study showed a potent anticancer activity towards Caco-2 and HCT-116 cells with low IC_50_ values ranging between 0.55 and 1.04 mM equivalent to 44.76 – 84.64 μg/ml. However, the substitution of Zn^2+^ ions by La^3+^ and/or Ce^3+^ ions enhanced the cytotoxic effect of ZnO NPs due to different factors, including the concentration and morphology of the NPs and level of ROS generation (Karthikeyan et al., 2019, Theivarasu and Indumathi, 2017). Shakir et al. (2016) showed that La-doped ZnO NPs exerted higher toxicity against different cancer cell lines than the pure sample treatment. Similarly, it was reported that Ce-doped ZnO NPs, fabricated via the co-precipitation method, induced a more cytotoxic effect against (A549) human lung cancer with lower IC_50_ values compared to the pure sample (Theivarasu and Indumathi, 2017). The authors attributed that to the increase of induced ROS production, resulting in apoptosis and cell death. The current results showed that ZnO-Ce NPs induced a more cytotoxic effect towards HCT-116 cells with low IC_50_ values and almost the same cytotoxic effect towards Caco-2 cells compared with the results obtained by Theivarasu and Indumathi. (2017). This enhancement in the anticancer potential of ZnO-Ce NPs could be attributed to the decrease in the particle size controlled using EDTA in the current study and the treated cell line. Based on the obtained results, EDTA significantly controlled the morphology of the prepared NPs, assured their stability, and enhanced their solubility in water, thus improving their biological efficiency.

## 5 Conclusion

The chemical co-precipitation technique allowed the successful synthesis of pure and lanthanides mono- and co-doped ZnO NPs with La^3+^ and/or Ce^3+^ ions, using EDTA as a capping agent. The wurtzite structure of ZnO NPs was validated by the XRD measurements and assured by the Reitveld refinements using the Maud program. Minor secondary phases of La_2_O_3_ and/or CeO_2_ were detected by the Maud program in the three doped patterns. Moreover, the morphology of ZnO NPs was greatly affected by the incorporation of La and/or Ce into the ZnO lattice and EDTA. The average particle size was reduced to a small-sized distorted hexagonal form with the appearance of two particle size distributions in the ZnO-LaCe sample. PL studies show the existence of several DL defects in the prepared NPs. This study showed that the prepared NPs exerted a significant cytotoxic effect on the two investigated human colon cancer cell lines and potent antibacterial efficiency toward the investigated bacteria. The IC_50_ values range between 0.48 and 1.58 mM. The current data showed that ZnO-Pure, ZnO-La, and ZnO-Ce samples induced more toxicity against HCT-116 cells than the Caco-2 cells. However, the ZnO-LaCe sample induced almost the same cytotoxic effect on both investigated cell lines. The ZnO-Pure sample showed the highest antibacterial activity towards the gram-negative bacteria among the prepared samples. Only the ZnO-LaCe sample showed an inhibitory effect against *Streptococcus intermedius*. The three doped samples enhanced the antibacterial effect of ZnO NPs toward *Staphylococcus aureus*, whereas only ZnO-Ce and ZnO-LaCe samples showed an inhibitory effect toward *Staphylococcus haemolyticus*. The present study showed that lanthanides mono- and co-doped ZnO NPs might have the potential as an auspicious antibacterial and anticancer agent. Future investigations are needed to assess the *in vivo* toxicity effect and to illustrate more about the mechanism of action of the fabricated NPs.

## Declaration of Competing Interest

The authors declare that they have no known competing financial interests or personal relationships that could have appeared to influence the work reported in this paper.

## References

[b0005] Abbott Chalew T.E., Schwab K.J. (2013). Toxicity of commercially available engineered nanoparticles to Caco-2 and SW480 human intestinal epithelial cells. Cell Biol. Toxicol..

[b0010] Ahmad M., Ahmed E., Zafar F., Khalid N.R., Niaz N.A., Hafeez A., Ikram M., Khan M.A., Hong Z. (2015). Enhanced photocatalytic activity of Ce-doped ZnO nanopowders synthesized by combustion method. J. Rare Earths.

[b0015] Al Bitar M., Khalil M., Awad R. (2022). Pure and lanthanum-doped zinc oxide nanoparticles: synthesis, characterization, and antibacterial activity. Appl. Phys. A.

[b0020] AL-Asady, Z.M., AL-Hamdani, A.H., Hussein, M.A., 2020. Study the optical and morphology properties of zinc oxide nanoparticles. Presented at the Presented at the 2nd International Conference of Materials Engineering & Science (IConMEAS 2019), Baghdad, Iraq, p. 020061. https://doi.org/10.1063/5.0000259.

[b0025] Aljohar, A.Y., Muteeb, G., Zia, Q., Siddiqui, S., Aatif, M., Farhan, M., Khan, Mohd.F., Alsultan, A., Jamal, A., Alshoaibi, A., Ahmad, E., Alam, M.W., Arshad, M., Ahamed, M.I., 2022. Anticancer effect of zinc oxide nanoparticles prepared by varying entry time of ion carriers against A431 skin cancer cells in vitro. Front. Chem. 10, 1069450. https://doi.org/10.3389/fchem.2022.1069450 10.3389/fchem.2022.1069450 PMC9751667 36531331

[b0030] Almoussawi M., Abdallah A.M., Habanjar K., Awad R. (2020). Effect of (Sm, Co) co-doping on the structure and electrical conductivity of ZnO nanoparticles. Mater. Res. Express.

[b0040] Azam A., Ahmed O., Khan H., Memic A. (2012). Antimicrobial activity of metal oxide nanoparticles against Gram-positive and Gram-negative bacteria: a comparative study. Int. J. Nanomed..

[b0045] Bitar Z., Isber S., Noureddine S., Bakeer D.-E.-S., Awad R. (2017). Synthesis, characterization, optical properties, and electron paramagnetic resonance for nano Zn_0.5_Co_0.5_Fe_2−x_Pr_x_O_4_. J. Supercond. Nov. Magn..

[b0050] Bomila R., Srinivasan S., Venkatesan A., Bharath B., Perinbam K. (2017). Structural, optical and antibacterial activity studies of Ce-doped ZnO nanoparticles prepared by wet-chemical method. Mater. Res. Innov..

[b0055] Bomila R., Suresh S., Srinivasan S. (2019). Synthesis, characterization and comparative studies of dual doped ZnO nanoparticles for photocatalytic applications. J. Mater. Sci. Mater. Electron..

[b0060] Boroumand Moghaddam A., Moniri M., Azizi S., Abdul Rahim R., Bin Ariff A., Navaderi M., Mohamad R. (2017). Eco-friendly formulated zinc oxide nanoparticles: induction of cell cycle arrest and apoptosis in the MCF-7 cancer cell line. Genes.

[b0065] Carofiglio M., Barui S., Cauda V., Laurenti M. (2020). Doped zinc oxide nanoparticles: synthesis, characterization and potential use in nanomedicine. Appl. Sci..

[b0070] Chandrasekaran P., Viruthagiri G., Srinivasan N. (2012). The effect of various capping agents on the surface modifications of sol–gel synthesised ZnO nanoparticles. J. Alloys Compd..

[b0075] Chen J.-L., Devi N., Li N., Fu D.-J., Ke X.-W. (2018). Synthesis of Pr-doped ZnO nanoparticles: their structural, optical, and photocatalytic properties. Chin. Phys. B.

[b0080] Dizaj S.M., Lotfipour F., Barzegar-Jalali M., Zarrintan M.H., Adibkia K. (2014). Antimicrobial activity of the metals and metal oxide nanoparticles. Mater. Sci. Eng. C.

[b0085] Dulta K., Koşarsoy Ağçeli G., Chauhan P., Jasrotia R., Chauhan P.K. (2021). A novel approach of synthesis zinc oxide nanoparticles by Bergenia ciliata Rhizome extract: antibacterial and anticancer potential. J. Inorg. Organomet. Polym. Mater..

[b0090] El-Belely E.F., Farag M.M.S., Said H.A., Amin A.S., Azab E., Gobouri A.A., Fouda A. (2021). Green synthesis of zinc oxide nanoparticles (ZnO-NPs) using Arthrospira platensis (Class: Cyanophyceae) and evaluation of their biomedical activities. Nanomaterials.

[b0095] Farhat S., Rekaby M., Awad R. (2018). Synthesis and characterization of Er-doped nano ZnO samples. J. Supercond. Nov. Magn..

[b0100] Fifere N., Airinei A., Dobromir M., Sacarescu L., Dunca S.I. (2021). Revealing the effect of synthesis conditions on the structural, optical, and antibacterial properties of cerium oxide nanoparticles. Nanomaterials.

[b0105] Flores-Carrasco G., Rodríguez-Peña M., Urbieta A., Fernández P., Rabanal M.E. (2021). ZnO nanoparticles with controllable ce content for efficient photocatalytic degradation of MB synthesized by the polyol method. Catalysts.

[b0110] Gnanam S., Ashokkumar R., SenthilKannan K. (2019). Antimicrobial activity of the novel metal oxide nanoparticles against selected human pathogenic bacteria. IOP Conf. Ser. Mater. Sci. Eng..

[b0115] Goel S., Sinha N., Yadav H., Joseph A.J., Kumar B. (2017). Experimental investigation on the structural, dielectric, ferroelectric and piezoelectric properties of La doped ZnO nanoparticles and their application in dye-sensitized solar cells. Phys. E Low-Dimens. Syst. Nanostruct..

[b0120] Hamida R.S., Abdelmeguid N.E., Ali M.A., Bin-Meferij M.M., Khalil M.I. (2020). Synthesis of silver nanoparticles using a novel cyanobacteria Desertifilum sp. extract: their antibacterial and cytotoxicity effects. Int. J. Nanomed..

[b0125] Hamida R.S., Ali M.A., Goda D.A., Khalil M.I., Redhwan A. (2020). Cytotoxic effect of green silver nanoparticles against ampicillin-resistant *Klebsiella pneumoniae*. RSC Adv..

[b0130] Hamida R.S., Ali M.A., Goda D.A., Khalil M.I., Al-Zaban M.I. (2020). Novel Biogenic Silver Nanoparticle-Induced Reactive Oxygen Species Inhibit the Biofilm Formation and Virulence Activities of Methicillin-Resistant Staphylococcus aureus (MRSA) Strain. Front. Bioeng. Biotechnol..

[b0135] Hammoudi N., Riaz Ahmed K.B., Garcia-Prieto C., Huang P. (2011). Metabolic alterations in cancer cells and therapeutic implications. Chin. J. Cancer.

[b0140] Iqbal J., Liu X., Zhu H., Wu Z.B., Zhang Y., Yu D., Yu R. (2009). Raman and highly ultraviolet red-shifted near band-edge properties of LaCe-co-doped ZnO nanoparticles. Acta Mater..

[b0145] Iseppi R., Tardugno R., Brighenti V., Benvenuti S., Sabia C., Pellati F., Messi P. (2020). Phytochemical composition and *in vitro* antimicrobial activity of essential oils from the Lamiaceae family against Streptococcus agalactiae and Candida albicans biofilms. Antibiotics.

[b0150] Jam N., Hajimohammadi R., Gharbani P., Mehrizad A. (2022). Antibacterial activity of Punica granatum L. and Areca nut (P.A) combined extracts against some food born pathogenic bacteria. Saudi J. Biol. Sci..

[b0155] Jang Y., Lee N., Kim J., Park Y., Piao Y. (2018). Shape-controlled synthesis of Au nanostructures using EDTA tetrasodium salt and their photothermal therapy applications. Nanomaterials.

[b0160] Jasim S.A., Saleh N.A. (2019). The cytotoxic effect of zinc oxide on colon cancer cell lines *in vitro*. Indian J. Public Health Res. Dev..

[b0165] Javed R., Usman M., Tabassum S., Zia M. (2016). Effect of capping agents: structural, optical and biological properties of ZnO nanoparticles. Appl. Surf. Sci..

[b0170] Jayachandraiah, C., Krishnaiah, G., Kumar, K.S., 2014. Ce induced structural and optical properties of Ce doped ZnO nanopartilces 6, 3378–3381

[b0175] Jeevanandam J., Barhoum A., Chan Y.S., Dufresne A., Danquah M.K. (2018). Review on nanoparticles and nanostructured materials: history, sources, toxicity and regulations. Beilstein J. Nanotechnol..

[b0180] Jiang J., Pi J., Cai J. (2018). The advancing of zinc oxide nanoparticles for biomedical applications. Bioinorg. Chem. Appl..

[b0185] Kamareddine F., Al Boukhari J., Awad R. (2020). Optoelectronic investigations of needle-shaped Zn _1–x_ Sn _x_ O nanoparticles synthesized by coprecipitation method. Phys. Scr..

[b0190] Karthikeyan M., Ahamed A.J., Kumar P.V. (2018). Facile green synthesis of LaCe co-doped ZnO nanoparticles and their structural, optical and antibacterial properties. J. Nanosci. Technol..

[b0195] Karthikeyan M., Ahamed A.J., Vijayakumar P., Karthikeyan C. (2018). Green synthesis of pure ZnO and La doped ZnO nanoparticles and their structural, optical and antibacterial studies. EJBPS.

[b0200] Karthikeyan M., Jafar Ahamed A., Karthikeyan C., Vijaya Kumar P. (2019). Enhancement of antibacterial and anticancer properties of pure and REM doped ZnO nanoparticles synthesized using Gymnema sylvestre leaves extract. SN Appl. Sci..

[b0205] Khan I., Saeed K., Khan I. (2019). Nanoparticles: properties, applications and toxicities. Arab. J. Chem..

[b0210] Kumar S., Sahare P.D. (2014). Gd^3+^ incorporated ZnO nanoparticles: a versatile material. Mater. Res. Bull..

[b0215] Labhane P.K., Sonawane G.H., Sonawane S.H. (2018). Influence of rare-earth metal on the zinc oxide nanostructures: application in the photocatalytic degradation of methylene blue and p-nitro phenol. Green Process. Synth..

[b0220] Lallo da Silva B., Abuçafy M.P., Berbel Manaia E., Oshiro Junior J.A., Chiari-Andréo B.G., Pietro R.C.R., Chiavacci L.A. (2019). Relationship between structure and antimicrobial activity of zinc oxide nanoparticles: an overview. Int. J. Nanomed..

[b0225] Lang J., Han Q., Yang J., Li C., Li X., Yang L., Zhang Y., Gao M., Wang D., Cao J. (2010). Fabrication and optical properties of Ce-doped ZnO nanorods. J. Appl. Phys..

[b0230] Lang J., Wang J., Zhang Q., Li X., Han Q., Wei M., Sui Y., Wang D., Yang J. (2016). Chemical precipitation synthesis and significant enhancement in photocatalytic activity of Ce-doped ZnO nanoparticles. Ceram. Int..

[b0235] Lee, S., Lin, M., Lee, A., Park, Y., 2017. Lanthanide-Doped Nanoparticles for Diagnostic Sensing. Nanomaterials 7, 411. https://doi.org/10.3390/nano7120411 10.3390/nano7120411 PMC5746901 29168770

[b0240] Limaye M.V., Singh S.B., Das R., Poddar P., Kulkarni S.K. (2011). Room temperature ferromagnetism in undoped and Fe doped ZnO nanorods: microwave-assisted synthesis. J. Solid State Chem..

[b0245] Majeed S., Danish M., Ismail M.H.B., Ansari M.T., Ibrahim M.N.M. (2019). Anticancer and apoptotic activity of biologically synthesized zinc oxide nanoparticles against human colon cancer HCT-116 cell line- *in vitro* study. Sustain. Chem. Pharm..

[b0250] Manikandan A., Manikandan E., Meenatchi B., Vadivel S., Jaganathan S.K., Ladchumananandasivam R., Henini M., Maaza M., Aanand J.S. (2017). Rare earth element (REE) lanthanum doped zinc oxide (La: ZnO) nanomaterials: synthesis structural optical and antibacterial studies. J. Alloys Compd..

[b0255] Mishra S.K., Srivastava R.K., Prakash S.G., Yadav R.S., Panday A.C. (2010). Photoluminescence and photoconductive characteristics of hydrothermally synthesized ZnO nanoparticles. Opto-Electron. Rev..

[b0260] Morkoç H., Özgür Ü. (2009).

[b0265] Nabeel A.I. (2020). Samarium enriches antitumor activity of ZnO nanoparticles via downregulation of CXCR4 receptor and cytochrome P450. Tumor Biol..

[b0270] Nguyen L.T.T., Nguyen L.T.H., Duong A.T.T., Nguyen B.D., Quang Hai N., Chu V.H., Nguyen T.D., Bach L.G. (2019). Preparation, characterization and photocatalytic activity of La-doped zinc oxide nanoparticles. Materials.

[b0275] Nithiananth, S., Velraj, G., 2016. FESEM, XRD and UV-Visible Spectroscopic Studies on Pure and EDTA Capped Zinc Oxide Nanoparticles by Wet Chemical Method 4.

[b0280] Obeid M.M., Jappor H.R., Al-Marzoki K., Al-Hydary I.A., Edrees S.J., Shukur M.M. (2019). Unraveling the effect of Gd doping on the structural, optical, and magnetic properties of ZnO based diluted magnetic semiconductor nanorods. RSC Adv..

[b0285] Pal M., Pal U., Jiménez J.M.G.Y., Pérez-Rodríguez F. (2012). Effects of crystallization and dopant concentration on the emission behavior of TiO2: Eu nanophosphors. Nanoscale Res. Lett..

[b0290] Pandey P., Kurchania R., Haque F.Z. (2015). Structural, diffused reflectance and photoluminescence study of cerium doped ZnO nanoparticles synthesized through simple sol–gel method. Optik.

[b0295] Patel K.N., Deshpande M.P., Chauhan K., Rajput P., Sathe V., Pandya S., Chaki S.H. (2017). Synthesis, structural and photoluminescence properties of nano-crystalline Cu doped NiO. Mater. Res. Express.

[b0300] Phuruangrat A., Dumrongrojthanath P., Yayapao O., Arin J., Thongtem S., Thongtem T. (2016). Photocatalytic activity of La-doped ZnO nanostructure materials synthesized by sonochemical method. Rare Met..

[b0305] Poornaprakash B., Poojitha P.T., Chalapathi U., Subramanyam K., Park S.-H. (2016). Synthesis, structural, optical, and magnetic properties of Co doped, Sm doped and Co+Sm co-doped ZnS nanoparticles. Phys. E Low-Dimens. Syst. Nanostruct..

[b0310] Ren M., Zheng X., Gao H., Jiang A., Yao Y., He W. (2022). Nanomedicines targeting metabolism in the tumor microenvironment. Front. Bioeng. Biotechnol..

[b0315] Sanaeimehr Z., Javadi I., Namvar F. (2018). Antiangiogenic and antiapoptotic effects of green-synthesized zinc oxide nanoparticles using Sargassum muticum algae extraction. Cancer Nanotechnol..

[b0320] Shahzad K., Mushtaq S., Akhtar S., Yaseen K., Amin F., Ali Z. (2019). Effect of lanthanum substitution on shape and cytotoxicity of zinc oxide (La_x_Zn_1−x_O) nano-colloids. Mater. Res. Express.

[b0325] Shakir, M., Faraz, Mohd., Sherwani, Mohd.A., Al-Resayes, S.I., 2016. Photocatalytic degradation of the Paracetamol drug using Lanthanum doped ZnO nanoparticles and their *in-vitro* cytotoxicity assay. J. Lumin. 176, 159–167. https://doi.org/10.1016/j.jlumin.2016.03.027

[b0330] Sharma D., Jha R. (2017). Analysis of structural, optical and magnetic properties of Fe/Co co-doped ZnO nanocrystals. Ceram. Int..

[b0335] Suwanboon S., Amornpitoksuk P., Sukolrat A., Muensit N. (2013). Optical and photocatalytic properties of La-doped ZnO nanoparticles prepared via precipitation and mechanical milling method. Ceram. Int..

[b0340] Tang X., Choo E.S.G., Li L., Ding J., Xue J. (2010). Synthesis of ZnO nanoparticles with tunable emission colors and their cell labeling applications. Chem. Mater..

[b0345] Theivarasu C., Indumathi T. (2017). Effect of Ce^3+^ metal ions on the antibacterial and anticancer activity of zinc oxide nanoparticles prepared by coprecipitation method. Asian J. Pharm. Clin. Res..

[b0350] Ullah A., Saadullah M., Alvi F., Sherin L., Ali A., Shad N.A., Javed Y., Sajid M.M., Yasin G., Abbas W. (2022). Synergistic effect of silver doped ZnO nanomaterials enhances the anticancer potential against A459 lung cancer cells. J. King Saud Univ. - Sci..

[b0355] Vinardell M., Mitjans M. (2015). Antitumor activities of metal oxide nanoparticles. Nanomaterials.

[b0360] Wen, H., Wang, F., 2014. Lanthanide-Doped Nanoparticles, in: Nanocrystalline Materials. Elsevier, pp. 121–160. https://doi.org/10.1016/B978-0-12-407796-6.00004-X.

[b0365] Yasmeen S., Munawar T., Asghar M., Khan M.A., Hussain A., Iqbal F. (2020). Synthesis and photocatalytic study of Zn_0.90_Co_0.10_O and Zn_0.90_Co_0.05_M_0.05_O (M = Ca, Ba, Cr, Pb) nanocrystals: structural, optical and electrical investigations. J. Mater. Res. Technol..

[b0370] Yi Y., Zhang Y., Wang Y., Shen L., Jia M., Huang Y., Hou Z., Zhuang G. (2014). Ethylenediaminetetraacetic acid as capping ligands for highly water-dispersible iron oxide particles. Nanoscale Res. Lett..

