# Supplementary material for: Characterization and evaluation of the therapeutic benefits of pure and lanthanides mono- and co-doped zinc oxide nanoparticles
Source: Saudi J Biol Sci. 2023 Feb 23;30(4):103608. doi: 10.1016/j.sjbs.2023.103608 (PMC10009547; doi:10.1016/j.sjbs.2023.103608)
Supplement: Supplementary data 1 [file mmc1.docx]

# Supplementary Figure Captions

**Fig. S1** Methicillin Resistance test (a) *Staphylococcus aureus*, (b)*Staphylococcus haemolyticus* **Abbreviations:** Cipro, Ciprofloxacin; Doxy, Doxycycline; Amoxi, Amoxicillin. **Note:** The used concentration of Cipro, Doxy, and Amoxi is 12.5 mg/ml and Cefoxitin disk (30 µg).

**Fig. S2** Inhibitory effect of the three antibiotics dilutions; Ciprofloxacin, Amoxicillin, and Doxycycline toward six investigated bacteria. A stock of the three antibiotics solutions (12.5 mg/ml) was prepared (Al Bitar et al., 2022), three half-fold dilutions were carried out to use three different concentrations (6.25, 3.125 and 1.5625 mg/ml). The petri-dishes are labeled in decreasing order of concentration from 1 to 4 corresponding to the range of 12.5- 1.5625 mg/ml.

**Abbreviations:** Cipro, Ciprofloxacin; Doxy, Doxycycline; Amoxi, Amoxicillin.

**Fig. S3** Inhibitory effect of ZnO-Pure (Al Bitar et al., 2022), ZnO-La, ZnO-Ce and ZnO-LaCe NPs against *Escherichia coli*, *Citrobacter braakii*, and *Klebsiella pneumonia.* **Abbreviations:** Cipro, Ciprofloxacin; Doxy, Doxycycline; Amoxi, Amoxicillin.

**Fig. S4** Inhibitory effect of ZnO-Pure (Al Bitar et al., 2022), ZnO-La, ZnO-Ce and ZnO-LaCe NPs against *Staphylococcus aureus*, *Staphylococcus haemolyticus*, and *Streptococcus intermedius*. **Abbreviations:** Cipro, Ciprofloxacin; Doxy, Doxycycline; Amoxi, Amoxicillin.

**Fig. S5** MIC determination at an OD value of 590 nm. i) ZnO-Pure (a) *Escherichia coli*, (b) *Klebsiella pneumonia*, (c) *Staphylococcus aureus*, and (d) *Citrobacter braakii*. ii) ZnO-La (a) *Escherichia coli*, (b) *Klebsiella pneumonia*, and (c) *Staphylococcus aureus*. **Abbreviation:** MIC, minimum inhibitory concentration; OD, optical density.

**Fig. S6** MIC determination at an OD value of 590 nm. i) ZnO-Ce (a) *Escherichia coli*, (b) *Klebsiella pneumonia*, (c) *Citrobacter braakii*, (d) *Staphylococcus aureus*, and (e) *Staphylococcus haemolyticus*. ii)ZnO-LaCe (a) *Citrobacter braakii*, (b) *Staphylococcus aureus*, (c) *Staphylococcus haemolyticus*, and (d) *Streptococcus intermedius*. **Abbreviation:** MIC, minimum inhibitory concentration; OD, optical density.

**Fig. S7** Effect of ZnO-Pure, ZnO-La, ZnO-Ce and ZnO-LaCe NPs on the proliferation of Caco-2 and HCT-116 cells. These results were expressed as the cell viability (%) versus the Nps’ concentration (mM). MTT assay was performed to detect the living cells after 24 - 48 hours, respectively.

**Fig. S8** Morphological changes induced by ZnO-Pure NPs with varying concentrations on Caco-2 and HCT-116 cells after 24 - 48 hours using the Inverted Microscope with $\times$ 200 magnification with scale bar value 100 µm.

**Fig. S9** Morphological changes induced by ZnO-La NPs with varying concentrations on Caco-2 and HCT-116 cells after 24 - 48 hours using the Inverted Microscope with $\times$ 200 magnification with scale bar value 100 µm.

**Fig. S10** Morphological changes induced by ZnO-Ce NPs with varying concentrations on Caco-2 and HCT-116 cells after 24 - 48 hours using the Inverted Microscope with $\times$ 200 magnification with scale bar value 100 µm.

**Fig. S11** Morphological changes induced by ZnO-LaCe NPs with varying concentrations on Caco-2 and HCT-116 cells after 24 - 48 hours using the Inverted Microscope with $\times$ 200 magnification with scale bar value 100 µm.

**Fig. S12 (a)** Caco-2 and (b) HCT-116 cells treated with ZnO-Pure, ZnO-La, ZnO-Ce and ZnO-LaCe NPs for 24 - 48 hours and then stained with 0.5% crystal violet.

# Supplementary Figures


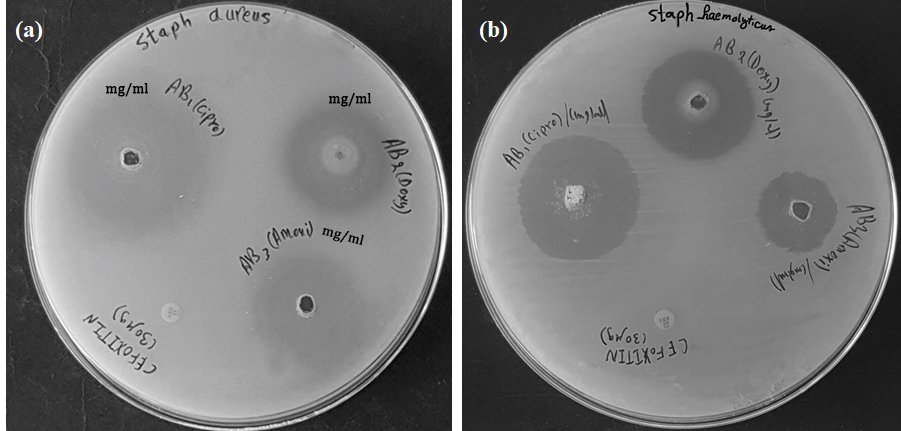


Fig. S1

**
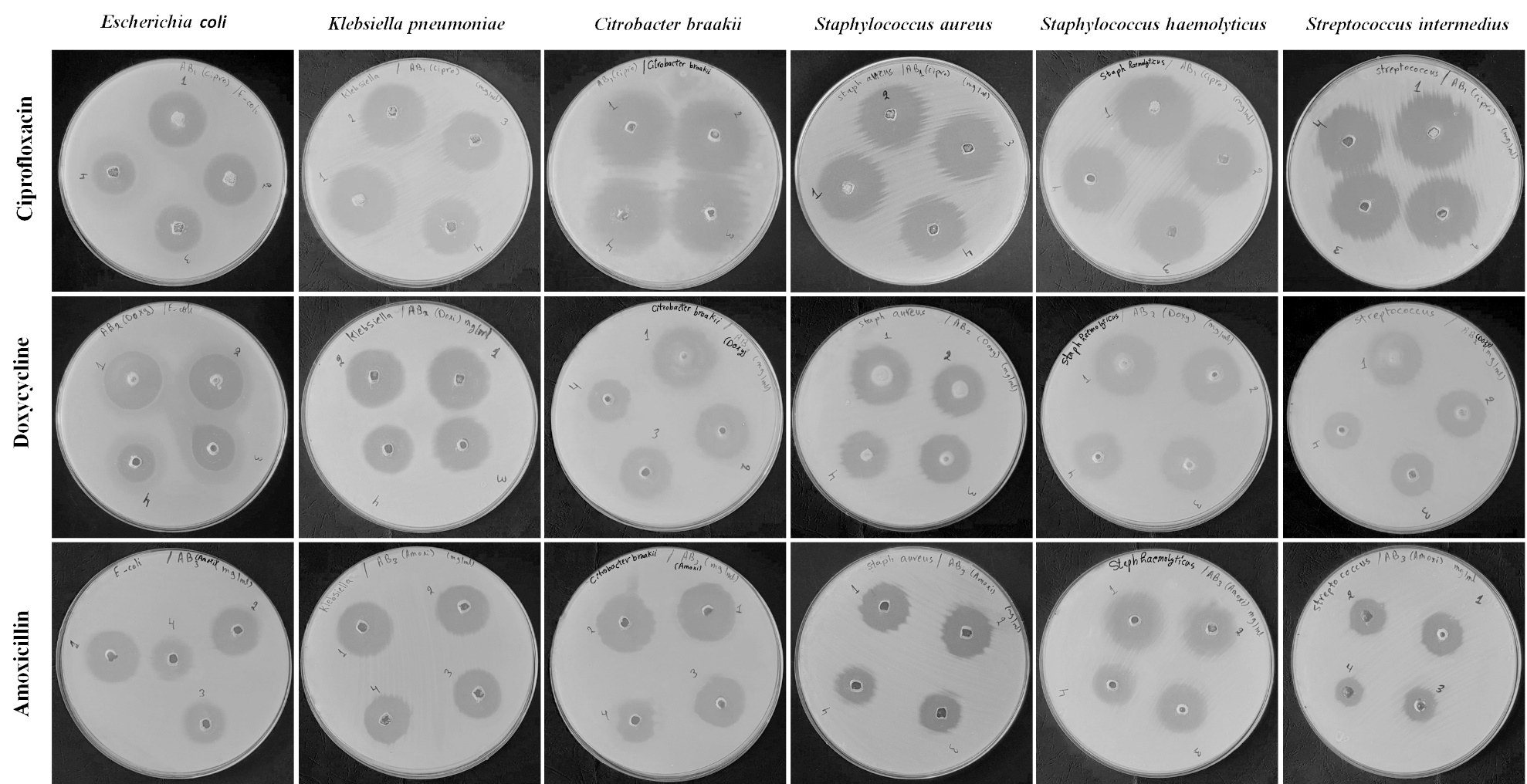
**

Fig. S2


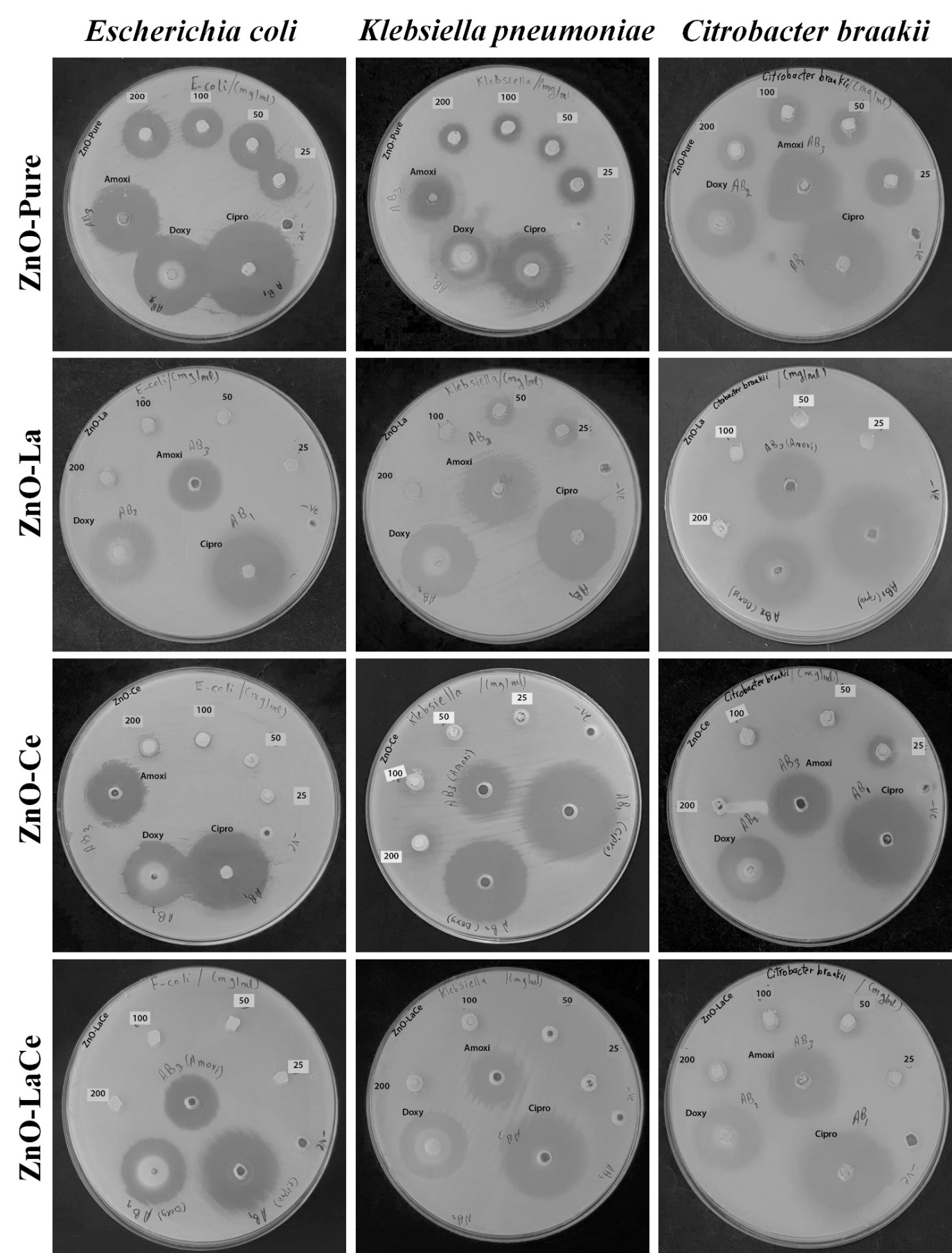


**Fig. S3**

**
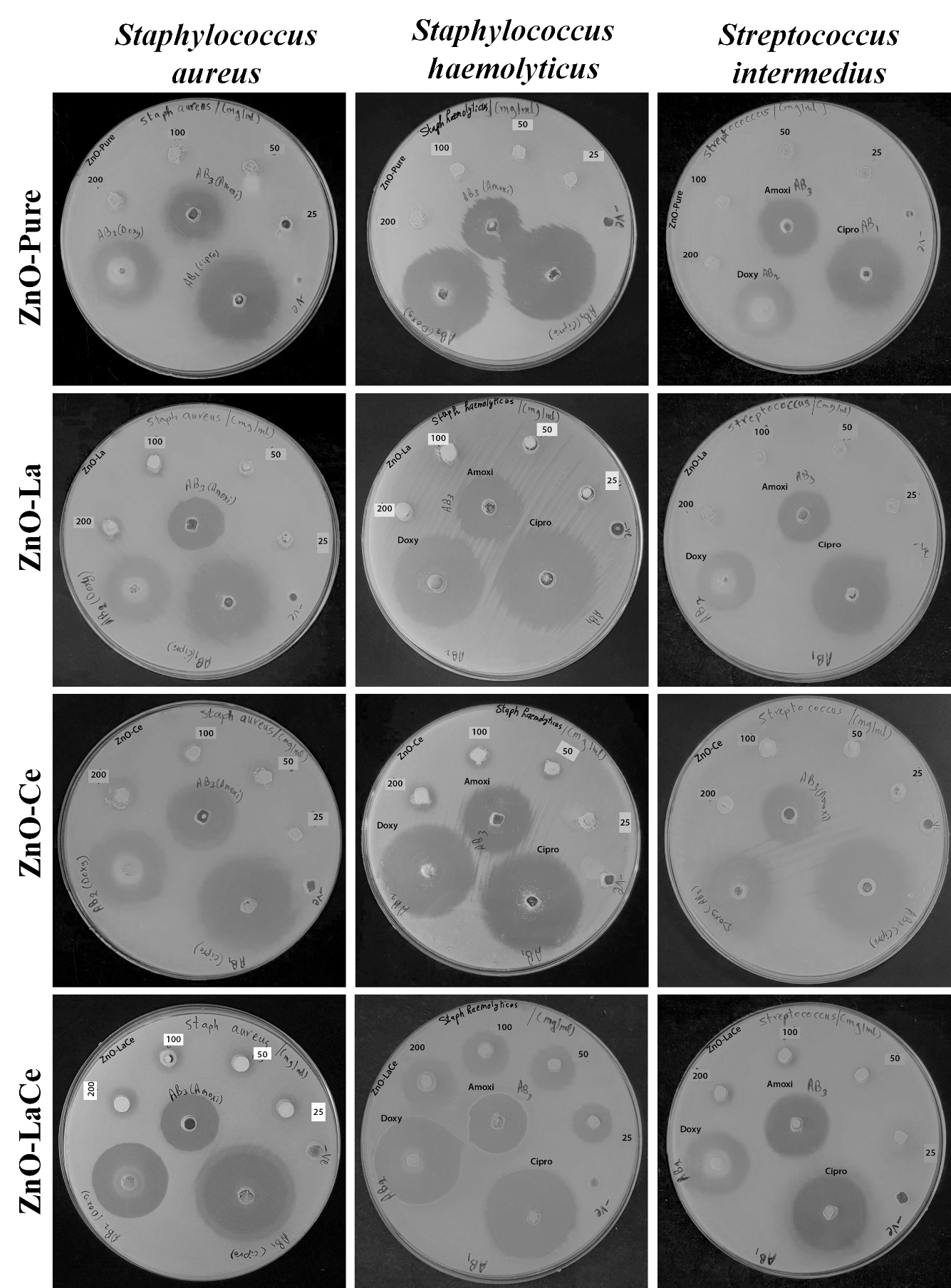
**

**Fig. S4**

**Fig. S5**

**Fig. S6**

Fig. S7

**
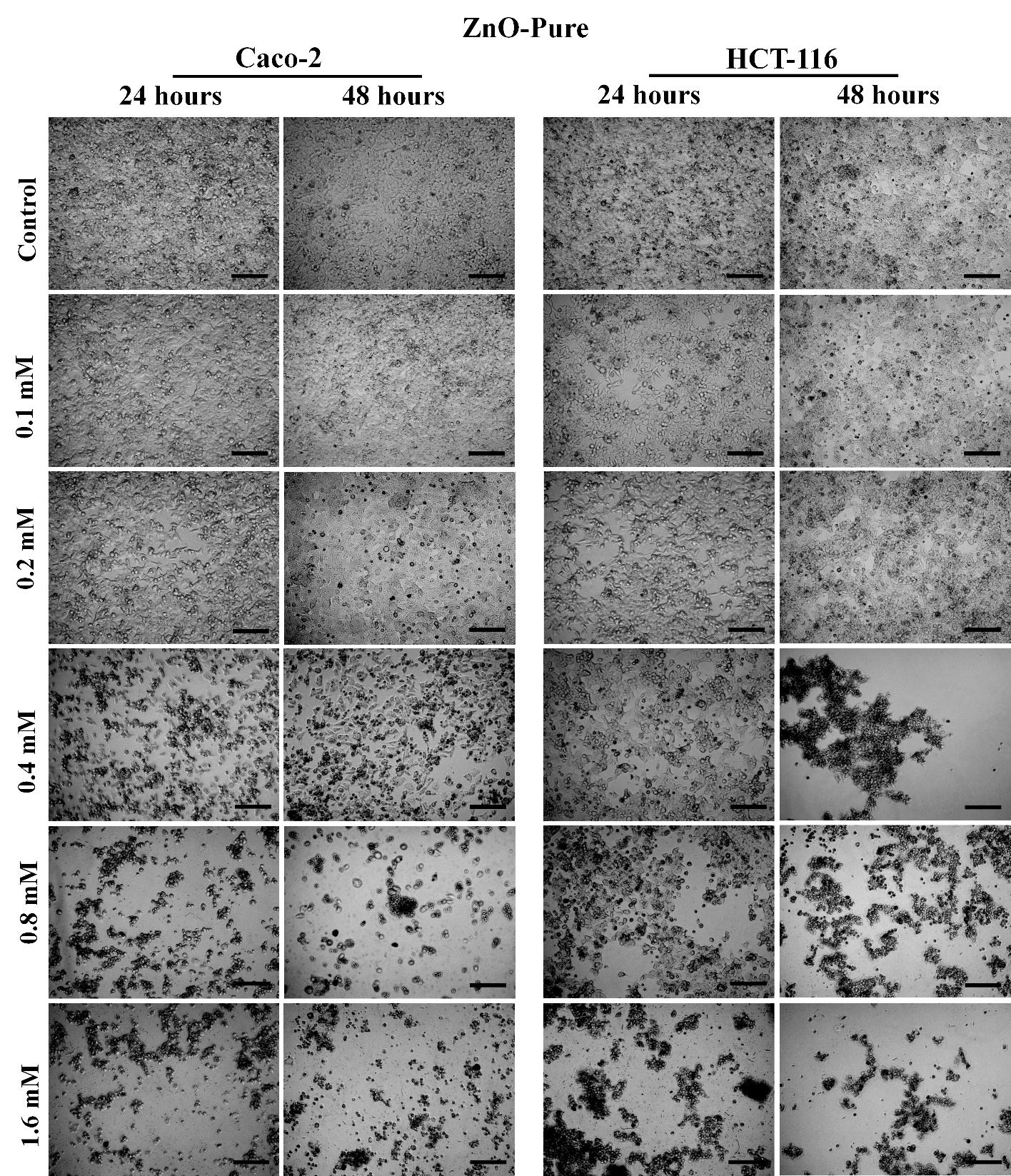
**

Fig. S8


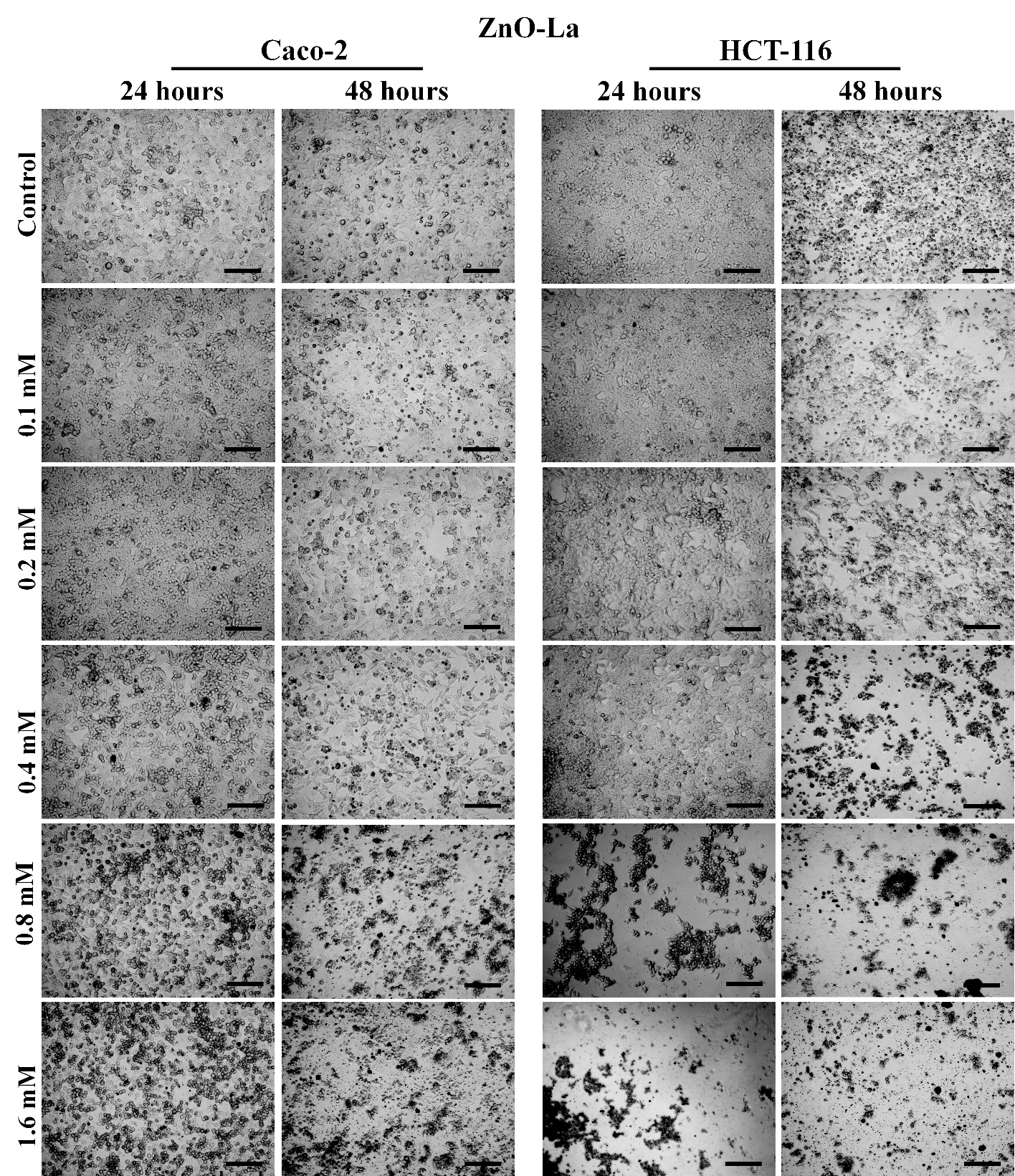


**Fig. S9**

**
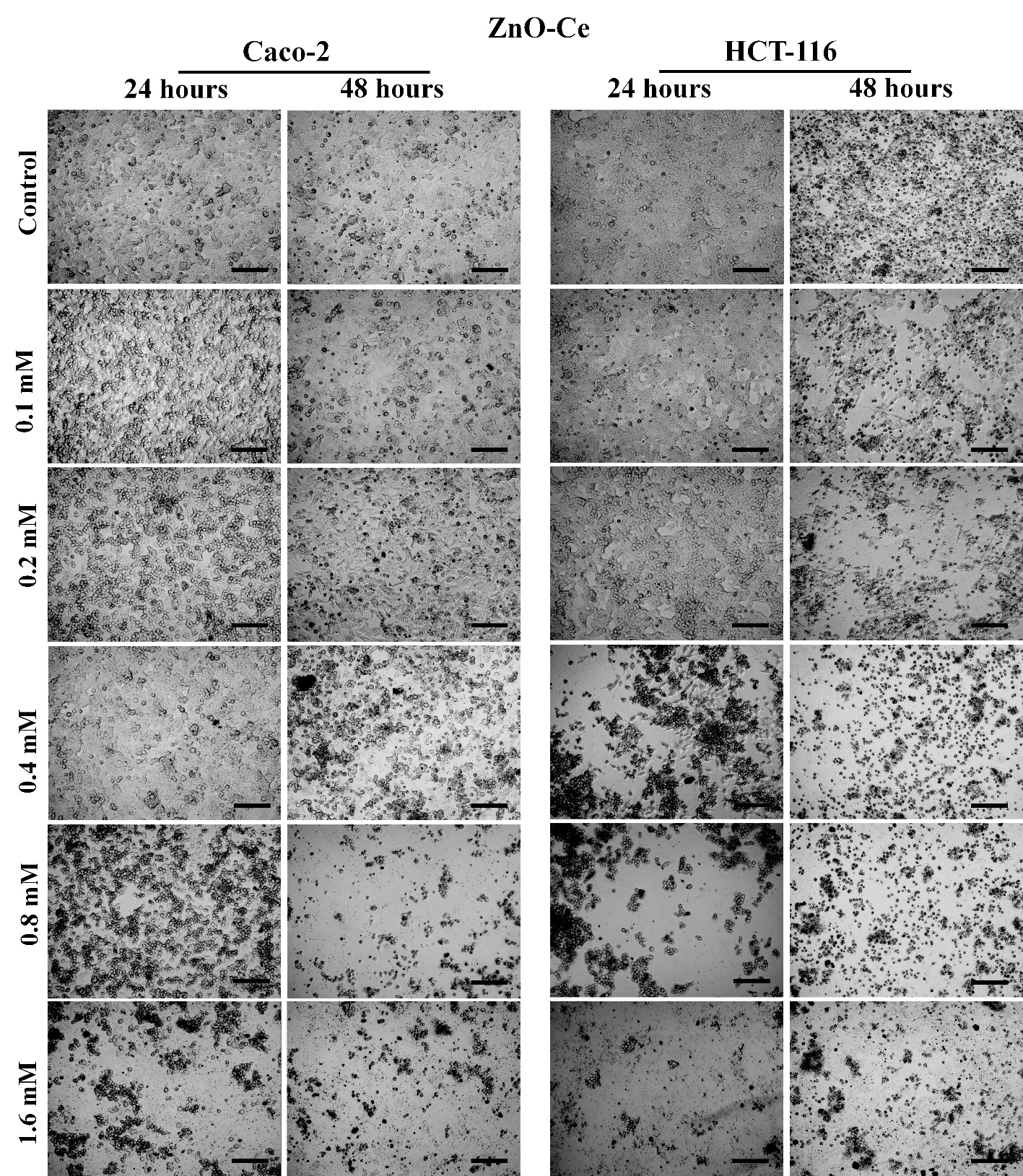
**

Fig. S10

**
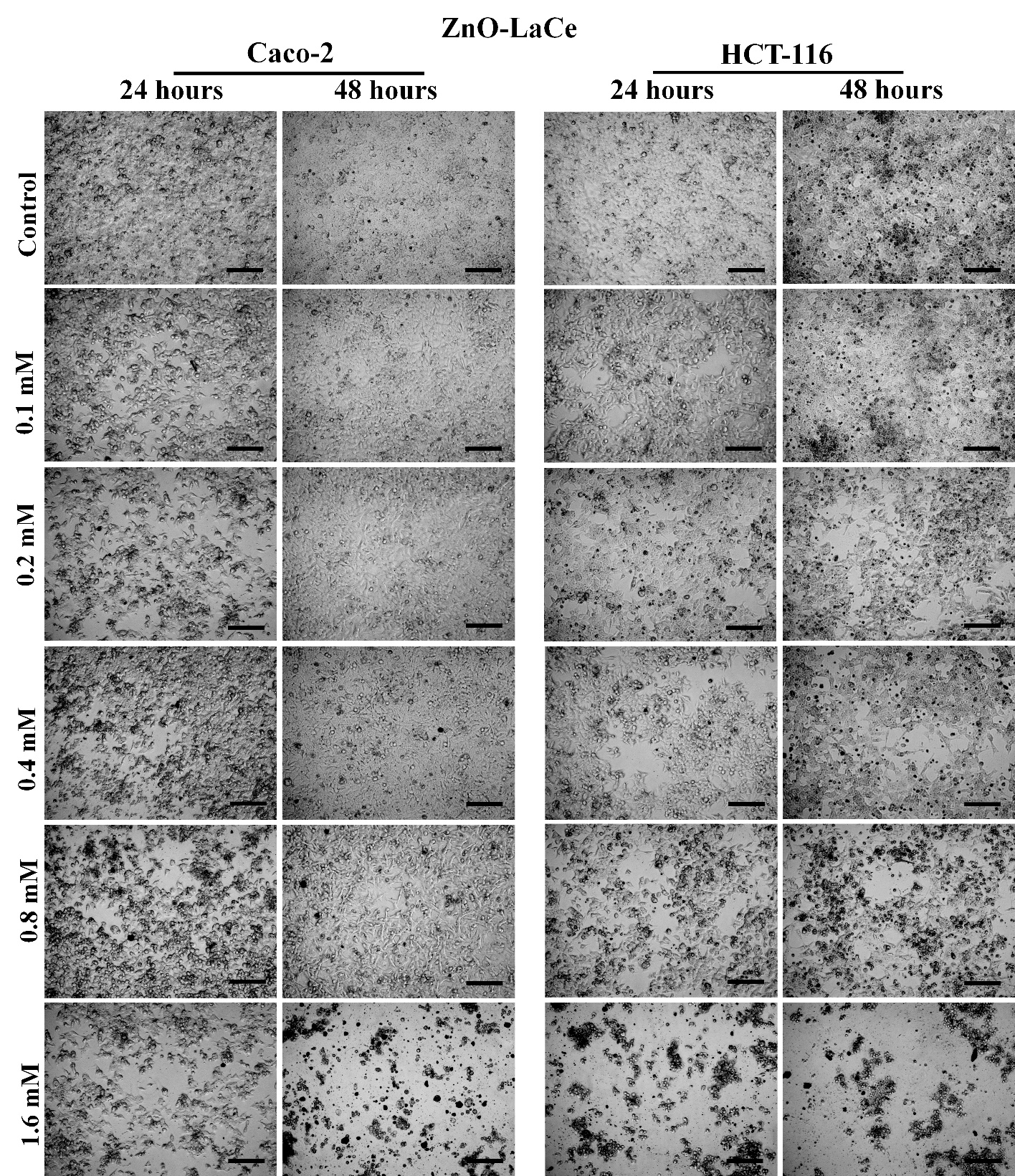
**

Fig. S1 1

**
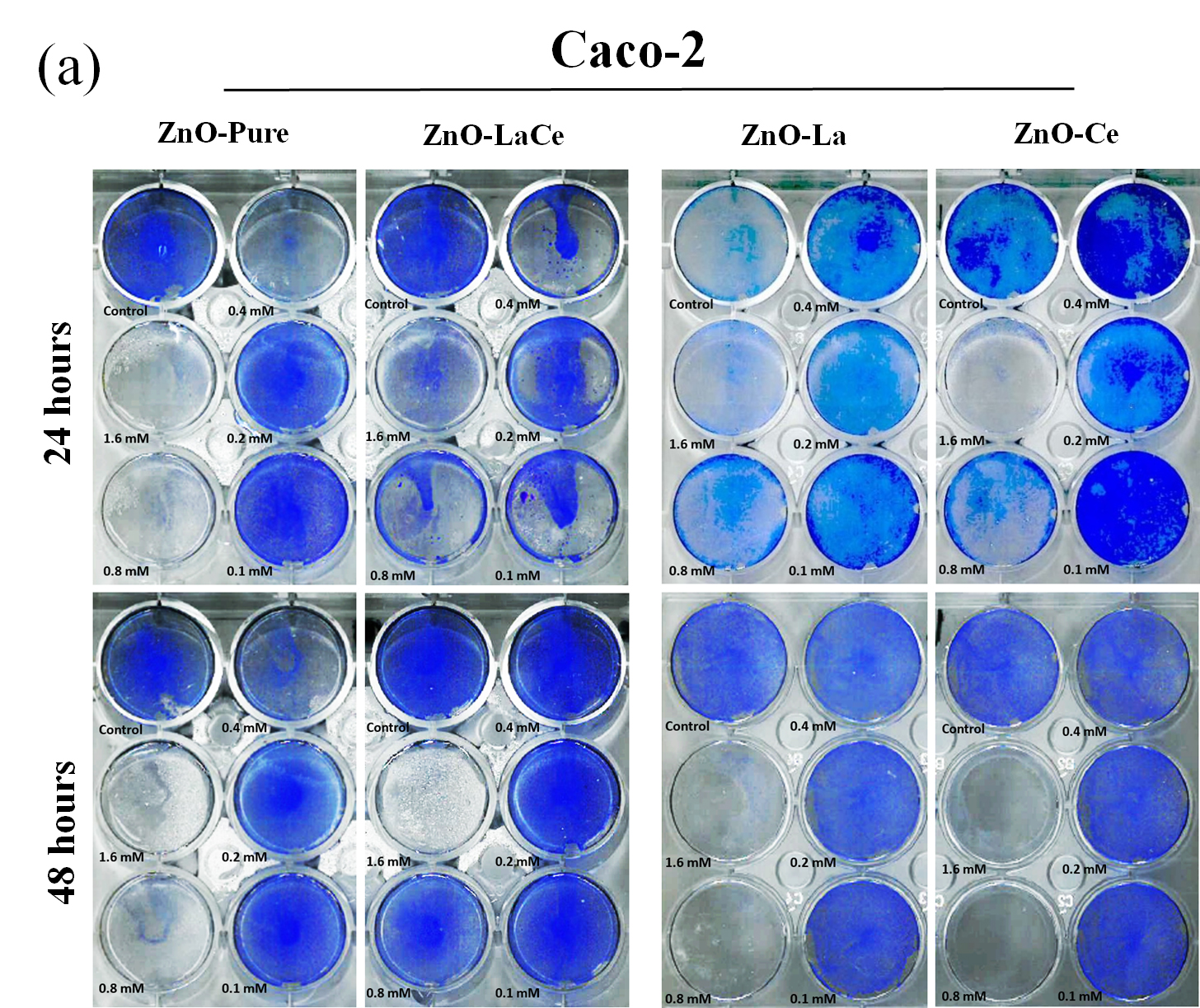
**

**Fig. S12-(a)**

**
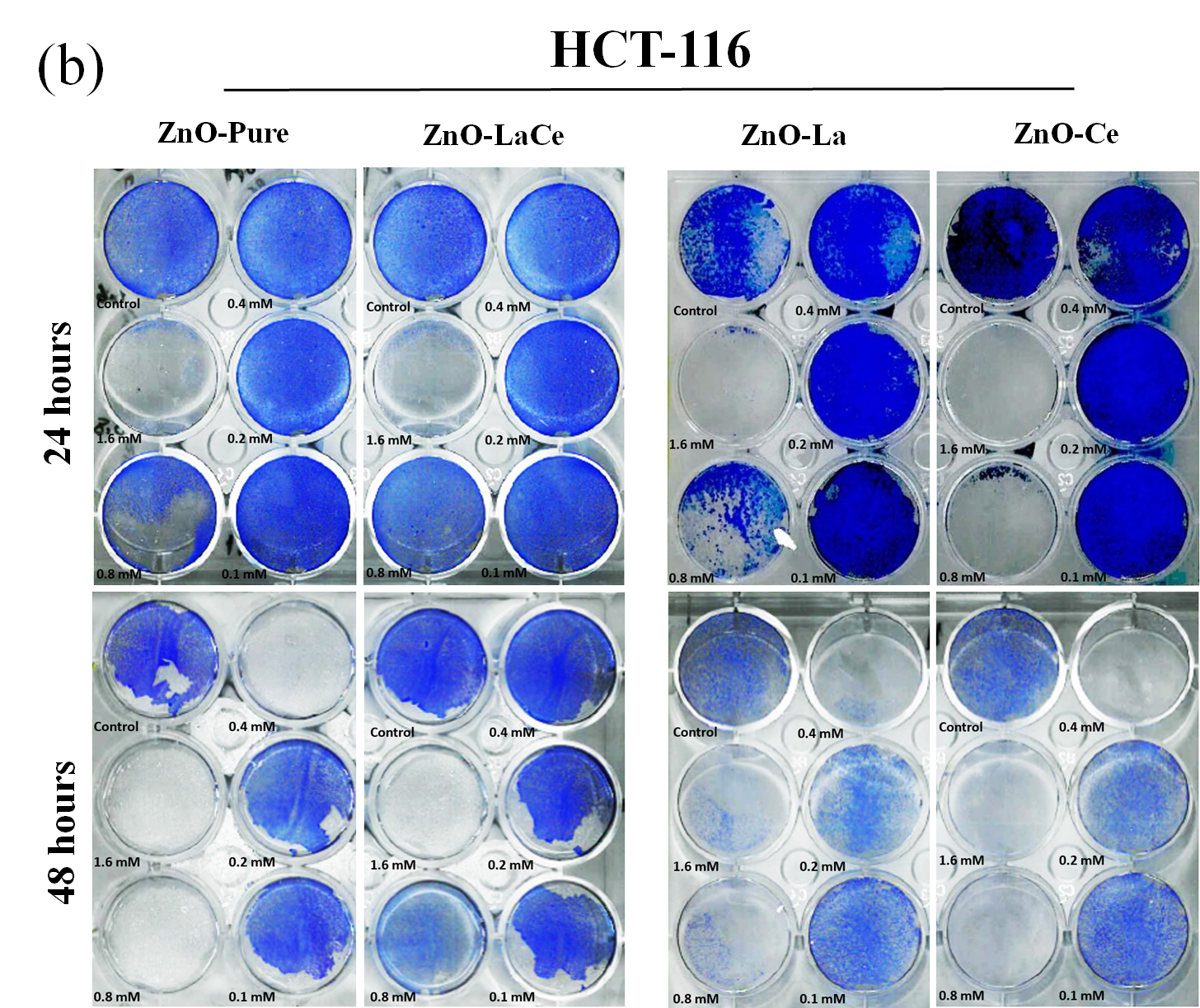
**

**Fig. S12-(b)**

# Supplementary Tables Captions

Table S1. Evaluation of the Inhibitory effect of ZnO-Pure (Al Bitar et al., 2022), ZnO-La, ZnO-Ce and ZnO-LaCe NPs, Ciprofloxacin, Amoxicillin, and Doxycycline using the agar well diffusion assay.

**Table S2.** The obtained confidence interval, *p* values, and the levels of significance of ZnO-Pure and ZnO-La NPs

**Table S3.** The obtained confidence interval, *p* values, and the levels of significance of ZnO-Ce NPs

**Table S4.** The obtained confidence interval, *p* values, and the levels of significance of ZnO-LaCe NPs

# List of Supplematray Tables **Table S1**

| Bacteria  Nanoparticles | | | *Escherichia coli* | *Klebsiella pneumoniae* | *Citrobacter braakii* | *Staphylococcus aureus* | *Staphylococcus haemolyticus* | *Streptococcus intermedius* |
| --- | --- | --- | --- | --- | --- | --- | --- | --- |
| Sample | Conc.(mg/ml) | | ZO$I\pm\mathrm{SEM}$ | | | | | |
| ZnO-Pure | 25 | | 20.43$\pm$0.16 | 17.29$\pm$0.32 | 24.42 $\pm$0.45 | 8.31$\pm$ 0.04 | 0 | 0 |
|  | 50 | | 21.42 $\pm$0.16 | 14.17 $\pm$ 0.14 | 19.07$\pm$ 0.05 | 8.65 $\pm$0.24 | 0 | 0 |
|  | 100 | | 23.06 $\pm$0.22 | 14.11 $\pm$ 0.15 | 19.04 $\pm$ 0.43 | 9.13 $\pm$0.15 | 0 | 0 |
|  | 200 | | 24.53$\pm$ 0.44 | 14.00 $\pm$0.12 | 18.73$\pm$ 0.23 | 10.29 $\pm$0.19 | 0 | 0 |
| ZnO-La | 25 | | 7.77 $\pm$0.24 | 15.06 $\pm$0.30 | 0 | 8.86 $\pm$0.20 | 0 | 0 |
|  | 50 | | 8.47 $\pm$0.15 | 14.86 $\pm$ 0.37 | 0 | 9.49 $\pm$0.39 | 0 | 0 |
|  | 100 | | 9.08 $\pm$0.27 | 10.00$\pm$ 0.23 | 0 | 11.14$\pm$ 0.24 | 0 | 0 |
|  | 200 | | 10.47$\pm$0.04 | 9.80 $\pm$ 0.08 | 0 | 12.12 $\pm$ 0.27 | 0 | 0 |
| ZnO-Ce | 25 | | 7.55 $\pm$0.18 | 9.36 $\pm$ 0.13 | 15.42$\pm$ 0.29 | 9.22 $\pm$ 0.11 | 8.22 $\pm$0.24 | 0 |
|  | 50 | | 7.61 $\pm$ 0.15 | 9.77 $\pm$ 0.17 | 10.96$\pm$ 0.27 | 14.72 $\pm$0.21 | 10.19 $\pm$ 0.22 | 0 |
|  | 100 | | 8.43 $\pm$0.18 | 11.41 $\pm$ 0.10 | 10.44$\pm$0.18 | 15.92 $\pm$0.22 | 11.93 $\pm$0.26 | 0 |
|  | 200 | | 11.45 $\pm$ 0.17 | 14.38$\pm$ 0.03 | 9.72$\pm$0.33 | 19.81 $\pm$ 0.19 | 13.66 $\pm$ 0.40 | 0 |
| ZnO-LaCe | 25 | | 0 | 0 | 8.50 $\pm$0.13 | 10.82 $\pm$0.09 | 19.01 $\pm$ 0.35 | 8.03 $\pm$ 0.07 |
|  | 50 | | 0 | 0 | 11.35 $\pm$0.17 | 12.49 $\pm$ 0.14 | 20.95 $\pm$ 0.27 | 8.65 $\pm$0.22 |
|  | 100 | | 0 | 0 | 11.36$\pm$0.18 | 13.63 $\pm$ 0.08 | 22.57 $\pm$ 0.28 | 10.29 $\pm$ 0.33 |
|  | 200 | | 0 | 0 | 12.57 $\pm$0.25 | 15.85 $\pm$0.13 | 24.90 $\pm$ 0.43 | 11.47 $\pm$ 0.05 |
| Ciprofloxacin | | 1.5625 | 28.35 $\pm$0.14 | 33.00 $\pm$ 0.26 | 40.46 $\pm$ 0.45 | 38.03 $\pm$ 0.16 | 38.53 $\pm$ 0.25 | 37.24 $\pm$ 0.13 |
|  |  | 3.125 | 34.08 $\pm$0.54 | 35.93 $\pm$ 0.27 | 42.86 $\pm$ 0.64 | 38.96 $\pm$ 0.13 | 40.62 $\pm$ 0.25 | 39.04 $\pm$ 0.22 |
|  |  | 6.25 | 37.51 $\pm$0.22 | 37.62$\pm$ 0.31 | 43.09 $\pm$ 0.68 | 39.82 $\pm$ 0.57 | 41.80 $\pm$ 0.24 | 40.74 $\pm$ 0.58 |
|  |  | 12.5 | 40.78 $\pm$ 0.82 | 38.26 $\pm$0.78 | 44.70 $\pm$ 0.36 | 41.03$\pm$ 0.52 | 47.41$\pm$0.84 | 42.02$\pm$ 1.05 |
| Doxycycline | | 1.5625 | 23.35 $\pm$ 0.13 | 29.41 $\pm$ 0.22 | 24.86 $\pm$ 0.33 | 27.23 $\pm$ 0.31 | 26.68 $\pm$ 0.26 | 22.06 $\pm$ 0.21 |
|  |  | 3.125 | 26.76$\pm$ 0.42 | 32.38 $\pm$ 0.26 | 30.40 $\pm$ 0.26 | 30.33 $\pm$ 0.31 | 32.16 $\pm$ 0.27 | 25.23 $\pm$ 0.31 |
|  |  | 6.25 | 32.12 $\pm$ 0.39 | 34.98 $\pm$ 0.18 | 32.45 $\pm$ 0.27 | 31.20 $\pm$ 0.32 | 33.13 $\pm$ 0.35 | 28.26 $\pm$ 0.27 |
|  |  | 12.5 | 33.36$\pm$ 0.58 | 35.08$\pm$ 0.69 | 34.90$\pm$ 0.26 | 35.14$\pm$0.50 | 40.61$\pm$0.98 | 31.67$\pm$ 0.37 |
| Amoxicillin | | 1.5625 | 22.70 $\pm$ 0.33 | 28.43 $\pm$ 0.26 | 24.47 $\pm$ 0.67 | 21.74 $\pm$ 0.67 | 24.94 $\pm$ 0.38 | 16.73 $\pm$ 0.33 |
|  |  | 3.125 | 24.71 $\pm$ 0.31 | 29.28 $\pm$ 0.36 | 29.12 $\pm$ 0.37 | 23.46 $\pm$ 0.30 | 29.12 $\pm$ 0.25 | 19.55 $\pm$ 0.26 |
|  |  | 6.25 | 28.02 $\pm$0.15 | 30.57$\pm$ 0.22 | 32.62 $\pm$ 0.36 | 28.49 $\pm$ 0.16 | 30.47 $\pm$ 0.25 | 21.54 $\pm$ 0.22 |
|  |  | 12.5 | 29.81 $\pm$0.66 | 31.84$\pm$ 0.73 | 33.78$\pm$ 0.39 | 28.69$\pm$ 0.75 | 32.39$\pm$0.93 | 25.76$\pm$ 0.76 |

**Notes:** The ZOI $\pm$ SEM (mm) in response to ZnO-Pure, ZnO-La, ZnO-Ce and ZnO-LaCe, Ciprofloxacin, Doxycycline, and Amoxicillin treatments toward the six investigated bacterial strains. The six bacteria were grown overnight on NA plates, and colonies formed were transferred to 0.85% normal saline for inoculum preparation.

| NPs | Concentration (mg/ml) | Bacteria | Confidence interval 95.00 % | *p* value | Level of significance |
| --- | --- | --- | --- | --- | --- |
| ZnO-Pure | 200*vs*.100 | *Escherichia coli* | 2.37 - 3.84 | $<$ 0.001 | *** |
|  | 200*vs*.50 |  | 0.73 - 2.20 | $<$ 0.001 | *** |
|  | 200*vs*.25 |  | 3.36 - 4.83 | $<$ 0.001 | *** |
|  | 100*vs*.50 |  | $-$2.37 - $-$0.89 | $<$ 0.001 | *** |
|  | 100*vs*.25 |  | 0.26 - 1.73 | 0.005 | ** |
|  | 50*vs*.25 |  | 1.89 - 3.36 | $<$ 0.001 | *** |
|  | 200*vs*.100 | *Klebsiella pneumonia* | $-$0.78 - 0.57 | 0.97 | ns |
|  | 200*vs*.50 |  | $-$0.84 - 0.51 | 0.91 | ns |
|  | 200*vs*.25 |  | $-$3.97 - $-$2.62 | $<$ 0.001 | *** |
|  | 100*vs*.50 |  | $-$0.73 - 0.62 | $>$ 0.99 | ns |
|  | 100*vs*.25 |  | $-$3.86 - $-$2.50 | $<$ 0.001 | *** |
|  | 50*vs*.25 |  | $-$3.81 - $-$2.41 | $<$ 0.001 | *** |
|  | 200*vs*.100 | *Staphylococcs aureus* | 0.39 - 1.92 | 0.002 | ** |
|  | 200*vs*.50 |  | 0.87 - 2.41 | $<$ 0.001 | *** |
|  | 200*vs*.25 |  | 1.21 - 2.74 | $<$ 0.001 | *** |
|  | 100*vs*.50 |  | $-$0.28 - 1.25 | 0.34 | ns |
|  | 100*vs*.25 |  | 0.05 - 1.56 | 0.03 | * |
|  | 50*vs*.25 |  | $-$0.43 - 1.10 | 0.64 | ns |
|  | 200*vs*.100 | *Citrobacter braakii* | $-$1.22 - 0.59 | 0.78 | ns |
|  | 200*vs*.50 |  | $-$1.24 - 0.56 | 0.73 | ns |
|  | 200*vs*.25 |  | $-$6.59 - $-$4.79 | $<$ 0.001 | *** |
|  | 100*vs*.50 |  | $-$0.93 - 0.87 | $>$ 0.99 | ns |
|  | 100*vs*.25 |  | $-$6.28 - $-$4.48 | $<$ 0.001 | *** |
|  | 50*vs*.25 |  | $-$6.25 - $-$4.45 | $<$ 0.001 | *** |
| ZnO-La | 200*vs*.100 | *Escherichia coli* | 0.57 - 2.21 | $<$ 0.001 | *** |
|  | 200*vs*.50 |  | 1.18 - 2.83 | $<$ 0.001 | *** |
|  | 200*vs*.25 |  | 1.88 - 3.52 | $<$ 0.001 | *** |
|  | 100*vs*.50 |  | $-$0.12 -1.35 | 0.13 | ns |
|  | 100*vs*.25 |  | 0.57 - 2.04 | $<$ 0.001 | *** |
|  | 50*vs*.25 |  | $-$0.04 -1.43 | 0.07 | ns |
|  | 200*vs*.100 | *Klebsiella pneumonia* | $-$0.78 - 0.48 | 0.87 | ns |
|  | 200*vs*.50 |  | $-$5.74 - $-$4.38 | $<$ 0.001 | *** |
|  | 200*vs*.25 |  | $-$5.94 - $-$4.58 | $<$ 0.001 | *** |
|  | 100*vs*.50 |  | $-$5.54 - $-$4.19 | $<$ 0.001 | *** |
|  | 100*vs*.25 |  | $-$5.75 - $-$4.39 | $<$ 0.001 | *** |
|  | 50*vs*.25 |  | $-$0.88 - 0.48 | 0.85 | ns |
|  | 200*vs*.100 | *Staphylococcs aureus* | 0.21 - 1.74 | 0.008 | ** |
|  | 200*vs*.50 |  | 1.87 - 3.40 | $<$ 0.001 | *** |
|  | 200*vs*.25 |  | 2.49 - 4.03 | $<$ 0.001 | *** |
|  | 100*vs*.50 |  | 0.89 - 2.43 | $<$ 0.001 | *** |
|  | 100*vs*.25 |  | 1.52 - 3.06 | $<$ 0.001 | *** |
|  | 50*vs*.25 |  | $-$0.14 - 1.39 | 0.14 | ns |

**Table S2**

| NPs | Concentration (mg/ml) | Bacteria | Confidence interval 95.00 % | *p* value | Level of significance |
| --- | --- | --- | --- | --- | --- |
| ZnO-Ce | 200*vs*.100 | *Escherichia coli* | 2.28 - 3.75 | $<$ 0.001 | *** |
|  | 200*vs*.50 |  | 3.11 - 4.58 | $<$ 0.001 | *** |
|  | 200*vs*.25 |  | 3.17 - 4.64 | $<$ 0.001 | *** |
|  | 100*vs*.50 |  | 0.09 - 1.56 | 0.02 | * |
|  | 100*vs*.25 |  | 0.15 - 1.62 | 0.01 | * |
|  | 50*vs*.25 |  | $-$0.68 -0.79 | $>$ 0.99 | ns |
|  | 200*vs*.100 | *Klebsiella pneumonia* | 2.30 - 3.65 | $<$ 0.001 | *** |
|  | 200*vs*.50 |  | 3.94 -5.29 | $<$ 0.001 | *** |
|  | 200*vs*.25 |  | 4.36 - 5.71 | $<$ 0.001 | *** |
|  | 100*vs*.50 |  | 0.97 - 2.32 | $<$ 0.001 | *** |
|  | 100*vs*.25 |  | 1.39 - 2.74 | $<$ 0.001 | *** |
|  | 50*vs*.25 |  | $-$0.26 -1.09 | 0.35 | ns |
|  | 200*vs*.100 | *Staphylococcs aureus* | 3.46 - 4.99 | $<$ 0.001 | *** |
|  | 200*vs*.50 |  | 4.33 - 5.86 | $<$ 0.001 | *** |
|  | 200*vs*.25 |  | 9.83 - 11.37 | $<$ 0.001 | *** |
|  | 100*vs*.50 |  | 0.10 - 1.64 | 0.02 | * |
|  | 100*vs*.25 |  | 5.61 - 7.14 | $<$ 0.001 | *** |
|  | 50*vs*.25 |  | 4.74 - 6.27 | $<$ 0.001 | *** |
|  | 200*vs*.100 | *Staphylococcs*  *haemolyticus* | 0.87 - 2.58 | $<$ 0.001 | *** |
|  | 200*vs*.50 |  | 2.62 - 4.33 | $<$ 0.001 | *** |
|  | 200*vs*.25 |  | 4.59 - 6.30 | $<$ 0.001 | *** |
|  | 100*vs*.50 |  | 0.90 - 2.61 | $<$ 0.001 | *** |
|  | 100*vs*.25 |  | 2.86 - 4.57 | $<$ 0.001 | *** |
|  | 50*vs*.25 |  | 1.11 - 2.82 | $<$ 0.001 | *** |
|  | 200*vs*.100 | *Citrobacter braakii* | $-$1.62 -0.19 | 0.16 | ns |
|  | 200*vs*.50 |  | $-$2.14 - $-$0.34 | 0.004 | ** |
|  | 200*vs*.25 |  | $-$6.60 - $-$4.80 | $<$ 0.001 | *** |
|  | 100*vs*.50 |  | $-$1.43 - 0.38 | 0.40 | ns |
|  | 100*vs*.25 |  | $-$5.89 - $-$4.08 | $<$ 0.001 | *** |
|  | 50*vs*.25 |  | $-$5.36 - $-$3.56 | $<$ 0.001 | *** |

**Table S3**

**Table S4**

| NPs | Concentration (mg/ml) | Bacteria | Confidence interval 95.00 % | *p* value | Level of significance |
| --- | --- | --- | --- | --- | --- |
| ZnO-LaCe | 200*vs*.100 | *Staphylococcs aureus* | 1.45 - 2.98 | $<$ 0.001 | *** |
|  | 200*vs*.50 |  | 2.59 - 4.13 | $<$ 0.001 | *** |
|  | 200*vs*.25 |  | 4.26 - 5.79 | $<$ 0.001 | *** |
|  | 100*vs*.50 |  | 0.38 - 1.91 | 0.02 | * |
|  | 100*vs*.25 |  | 2.04 - 3.58 | $<$ 0.001 | *** |
|  | 50*vs*.25 |  | 0.90 - 2.43 | $<$ 0.001 | *** |
|  | 200*vs*.100 | *Staphylococcs*  *haemolyticus* | 1.48 - 3.19 | $<$ 0.001 | *** |
|  | 200*vs*.50 |  | 3.09 - 4.81 | $<$ 0.001 | *** |
|  | 200*vs*.25 |  | 5.03 - 6.74 | $<$ 0.001 | *** |
|  | 100*vs*.50 |  | 0.76 - 2.47 | $<$ 0.001 | *** |
|  | 100*vs*.25 |  | 2.70 - 4.41 | $<$ 0.001 | *** |
|  | 50*vs*.25 |  | 1.08 - 2.79 | $<$ 0.001 | *** |
|  | 200*vs*.100 | *Citrobacter braakii* | 0.31 - 2.11 | 0.005 | ** |
|  | 200*vs*.50 |  | 0.32 - 2.13 | 0.004 | ** |
|  | 200*vs*.25 |  | 3.18 - 4.98 | $<$ 0.001 | *** |
|  | 100*vs*.50 |  | $-$0.89 - 0.91 | $>$ 0.99 | ns |
|  | 100*vs*.25 |  | 1.96 - 3.77 | $<$ 0.001 | *** |
|  | 50*vs*.25 |  | 1.95 - 3.75 | $<$ 0.001 | *** |
|  | 200*vs*.100 | *Streptococcus intermedius* | 0.79 - 1.57 | $<$ 0.001 | *** |
|  | 200*vs*.50 |  | 2.43 - 3.21 | $<$ 0.001 | *** |
|  | 200*vs*.25 |  | 3.06 - 3.83 | $<$ 0.001 | *** |
|  | 100*vs*.50 |  | 1.25 - 2.03 | $<$ 0.001 | *** |
|  | 100*vs*.25 |  | 1.87 - 2.65 | $<$ 0.001 | *** |
|  | 50*vs*.25 |  | 0.23 - 1.01 | $<$ 0.001 | *** |
